# An analytical workflow for accurate variant discovery in highly divergent regions

**DOI:** 10.1186/s12864-016-3045-z

**Published:** 2016-09-02

**Authors:** Shulan Tian, Huihuang Yan, Claudia Neuhauser, Susan L. Slager

**Affiliations:** 1 Division of Biomedical Statistics and Informatics, Department of Health Sciences Research, Mayo Clinic, 200 1st St SW, Rochester, MN 55905 USA; 2 Informatics Institute, University of Minnesota, Minneapolis, MN 55455 USA

**Keywords:** Alignment algorithm, Chronic lymphocytic leukemia, Exome sequencing, Human leukocyte antigen, Variant calling

## Abstract

**Background:**

Current variant discovery methods often start with the mapping of short reads to a reference genome; yet, their performance deteriorates in genomic regions where the reads are highly divergent from the reference sequence. This is particularly problematic for the human leukocyte antigen (HLA) region on chromosome 6p21.3. This region is associated with over 100 diseases, but variant calling is hindered by the extreme divergence across different haplotypes.

**Results:**

We simulated reads from chromosome 6 exonic regions over a wide range of sequence divergence and coverage depth. We systematically assessed combinations between five mappers and five callers for their performance on simulated data and exome-seq data from NA12878, a well-studied individual in which multiple public call sets have been generated. Among those combinations, the number of known SNPs differed by about 5 % in the non-HLA regions of chromosome 6 but over 20 % in the HLA region. Notably, GSNAP mapping combined with GATK UnifiedGenotyper calling identified about 20 % more known SNPs than most existing methods without a noticeable loss of specificity, with 100 % sensitivity in three highly polymorphic HLA genes examined. Much larger differences were observed among these combinations in INDEL calling from both non-HLA and HLA regions. We obtained similar results with our internal exome-seq data from a cohort of chronic lymphocytic leukemia patients.

**Conclusions:**

We have established a workflow enabling variant detection, with high sensitivity and specificity, over the full spectrum of divergence seen in the human genome. Comparing to public call sets from NA12878 has highlighted the overall superiority of GATK UnifiedGenotyper, followed by GATK HaplotypeCaller and SAMtools, in SNP calling, and of GATK HaplotypeCaller and Platypus in INDEL calling, particularly in regions of high sequence divergence such as the HLA region. GSNAP and Novoalign are the ideal mappers in combination with the above callers. We expect that the proposed workflow should be applicable to variant discovery in other highly divergent regions.

**Electronic supplementary material:**

The online version of this article (doi:10.1186/s12864-016-3045-z) contains supplementary material, which is available to authorized users.

## Background

Genetic variations in protein-coding genes play significant roles in many human diseases [1, 2] and are associated with the response to drug treatment [3]. Whole exome sequencing (WES) targets >95 % of the exons or approximately 1 % of the human genome [2, 4]. It has been widely used to identify causal variants [5], uncovering about 85 % of the causative mutations identified in Mendelian diseases [2, 6].

Multiple bioinformatics methods have been developed to identify variants from whole genome or exome sequencing data. The most dominant ones are based on the mapping of reads to a reference genome [7, 8], which often follow the GATK (Genome Analysis Tool Kit) Best Practices [9, 10]. The GATK Best Practices workflows recommend read mapping by Burrows-Wheeler Aligner (BWA), post-alignment processing, and then GATK variant calling. Compared to the hash-based mappers described below, the Burrows-Wheeler transform (BWT)-based mappers like BWA are faster but tend to be less sensitive [11–13]. They were developed for mapping reads to less divergent regions [11, 14].

Divergence level varies markedly across the human genome [15–17], which has a profound impact on the outcome of variant calling. While the bulk of the genome has only about 0.1 % divergence [12, 18], some regions are highly polymorphic [16, 19, 20]. The best example is the human leukocyte antigen (HLA) region on chromosome 6p21.3; this ~4-Mb region shows up to 10 % or higher local sequence divergence between haplotypes [18, 21, 22]. Most importantly, the HLA region is associated with over 100 diseases, predominantly autoimmune diseases [23], and also with drug response [24]. At such high divergence, the BWA mapping rate drops to a few percent [12, 13]. Thus, accurate identification of sequence variation in this region is clinically important but currently hindered by the extreme polymorphism.

A few mappers have been tailored to aligning reads to more divergent regions, such as GSNAP [14], NextGenMap [13], Novoalign (http://www.novocraft.com/) and Stampy [12]. They use 11- to 15-mer hash tables generated from the reference sequence. Among them, Novoalign and Stampy were found to be more accurate than BWA over a wide range of divergence [12, 25]. Stampy performed similarly as NextGenMap at 10 % divergence [13] but was superior to Novoalign at higher (10–15 %) divergence [12]. GSNAP is capable of mapping reads with multiple mismatches and/or long insertions and deletions (INDELs) [14]. The choice of an appropriate mapper has a big impact on variant calling [9, 26]. However, previous studies often used simulated data and their primary goal was to evaluate the overall sensitivity and accuracy of different mappers. Therefore, for these ‘variation-tolerant’ mappers, it remains less clear which one(s) may strongly enhance variant detection in highly divergent regions.

Several popular software packages are available for both single- and multi-sample variant calling, such as SAMtools [27], FreeBayes [28], GATK UnifiedGenotyper and HaplotypeCaller [9, 29]. Packages like GATK HaplotypeCaller, Scalpel [30] and Platypus [31] combine mapping and local assembly, which are particularly attractive for INDEL detection.

Numerous studies have compared the performance of variant callers on WES, without focusing on the highly divergent regions [32–34]. These studies used either BWA or ELAND2 as the mapper that has low sensitivity in mapping divergent reads. Moreover, they estimated variant detection accuracy by using single nucleotide polymorphism (SNP) sites genotyped on SNP arrays that primarily target common variants. A more recent study compared GATK HaplotypeCaller, GATK UnifiedGenotyper and SAMtools with Novoalign as the mapper [35]; however, the estimation of sensitivity was based on the high-confidence call set [36] that is known to have poor coverage in the HLA region. Two analyses revealed low concordance of different analytical pipelines on WES data, being only 27 % [37] or 37 % [30] for INDELs among three methods and less than 60 % for SNPs among five methods [37]. These two studies highlight the difficulties in obtaining high-quality variant calls from WES [31]. For the whole-genome sequencing, variant calling methods generally agree well with one another in about 90 % of the genome but show marked disagreement in the other ~10 % ‘difficult regions’ of low-complexity and segmental duplications [38]. Obviously, a better understanding of the factors leading to the low concordance among different approaches is critical for further optimization of variant discovery. Furthermore, overall genome-wide performance of a variant detection method may not reflect the local scenario in highly divergent regions.

We seek to develop a workflow for more accurate variant discovery from WES data, especially in highly divergent regions. By simulating reads from chromosome 6 exonic regions, we systematically evaluated five popular callers together with five mappers over a wide range of divergence level and coverage depth. Taking advantage of the existing call sets generated by whole genome and exome sequencing in NA12878, we verified the findings on two WES data in this well-studied CEU (Utah residents with Northern and Western European ancestry) sample. Our analysis revealed key factors impacting variant discovery accuracy and sensitivity. We identified the best mapper-caller combinations for variant detection in both HLA and non-HLA regions, and further demonstrated their excellence on WES data from a cohort of chronic lymphocytic leukemia (CLL) patients. Our strategies are particularly effective for WES and should be applicable to whole genome sequencing data as well.

## Methods

### Simulation of exome-seq reads

**Fig. 1:**
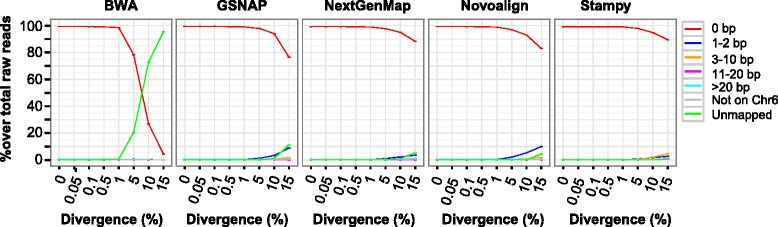
Mapping status of simulated reads. Eight datasets were simulated to 100x per-base coverage at seven divergence levels (0.05–15 %) or without introducing sequence variation (control). After mapping, the total reads were first broken into those mapping to chromosome 6, those mapping to other chromosomes (‘Not on Chr6’) and unmapped reads (‘Unmapped’). The reads mapping to chromosome 6 were then grouped into five clusters based on the distance (0, 1–2, 3–10, 11–20 and >20 bp) from their original locations to the mapping locations reported by a mapper

The variation level in the human genome is typically ~0.1 % [12] but can reach over 10 % in some extremely divergent loci like those located in the HLA region [18, 21]. Therefore, in the simulation we defined seven divergence levels between 0.05 and 15 % and a control with a zero percent divergence (Fig. 1). Here, divergence level represents the ratio of the number of permuted SNPs and INDELs over the total bases of the regions included in the simulation. We compiled 10,768 non-overlapping exonic regions of chromosome 6, from which 100-base paired-end reads were simulated to an average coverage of 100x using Dwgsim (Additional file 1: method 1). To investigate the impact of coverage depth on variant calling, we randomly sampled six subsets with coverage depth of 80x, 60x, 40x, 20x, 10x and 5x, respectively.

### Mapping simulated reads to the reference sequence

Five mappers were selected to align simulated reads against the hg19 reference sequence (Additional file 1: method 2), using the parameter settings in Additional file 2: Table S1. Specifically, we set the divergence level or substitution rate at 10 % for Stampy (−−substitutionrate = 0.1, default 0.001) and GSNAP (−−max-mismatches 0.1, default 0.1 for 100 bp reads and kmer = 13) and at 20 % for NextGenMap (−−min-identity 0.8, default 0.65). We used the default (no more than 2 mismatches in the first 32 bp) for mismatches in the ‘bwa aln’ command. Novoalign does not have the parameter for specifying the divergence level. The performance was measured on the basis of mapping rate and accuracy. The former was defined as the ratio of mapped reads over the total number of simulated reads, and the latter as the ratio of reads mapping back to their original locations over the total. To simplify both calculations, the two reads from each pair were treated as single-end reads without considering the pairing information. Four of the mappers (except Stampy) use ‘soft-clipping’, which skips unaligned terminal portion(s) from reads and only reports partial alignments between reads and the reference sequence. In estimating mapping accuracy we counted the number of soft-clipped bases shown in the CIGAR string and added it back to the reported mapping location. This adjusted mapping location was then compared to the original location from where a read was simulated. Mapping accuracy was estimated as the ratio of reads mapping back to the original start position over the total simulated reads.

### Variant calling from simulated data

To reduce erroneous calls, alignments were subjected to duplicate marking and local realignment by following the GATK Best Practices [9, 10], but without base quality score recalibration (Additional file 1: method 3). Five callers were selected, including GATK UnifiedGenotyper and HaplotypeCaller [9, 29], FreeBayes [28], SAMtools mpileup [27], and Platypus [31]. The command and parameter settings can be found in Additional file 2: Table S1. Parameters were selected to ensure comparability among different callers. For GATK UnifiedGenotyper and HaplotypeCaller, we set the minimum phred-scaled quality score of 20 (default 30) in variant calling and of 10 (default 30) in variant reporting. A minimum base quality score of 17 was used for four of the callers, except for GATK HaplotypeCaller that did not provide this parameter in GATK v 2.7–2 used in this study. Only variants with a quality score of at least 20 were used in the comparison. BEDTools was used to identify overlap between permuted and called INDELs [39]. The performance of individual methods was evaluated on the basis of sensitivity, precision rate, and overall genotype concordance (Additional file 1: method 3).

### Variant calling from NA12878 exome-seq data

To confirm the generality of the findings made from simulated reads, we tested the same mappers and callers on WES data from DNA sample NA12878. NA12878 is the first genome for which the reference genotype calls (‘high confident call set’) were generated by the Genome in a Bottle Consortium from 11 whole genome and three exome sequencing datasets [36]. The analysis integrates seven mappers and three callers. This call set covers ‘ordinary’ variants which are more readily to be identified, while the ‘difficult’ ones are largely excluded, mostly in regions of low-complexity, segmental duplications and structural variations [36]. In addition, three other call sets were generated by GATK HaplotypeCaller and two *de novo* assembly-based callers, Cortex and DISCOVAR, from 250-base paired sequencing of a PCR-free genomic library [38]. They focused more on difficult variants in regions of low-complexity and segmental duplications. The union of these four call sets (referred to as ‘public call set’) was treated as the ‘true’ variants. Since this public call set is compiled from variants identified through distinct analytical algorithms, multiple library preparation protocols and sequencing platforms, systematic bias toward a particular method should have been minimized in the assessment (Additional file 1: method 4).

Illumina, Inc. generated 12 replicates of 150 base-pair (bp) paired-end exome data in NA12878 (https://basespace.illumina.com/home/index). We downloaded two replicates, each with ~100x coverage on average. Considering that the simulated reads have a length of 100 bases, pairs with one or both reads shorter than 100 bases were filtered out, which excluded 5.1 and 5.6 % of the reads, respectively, from the two replicates. From the remainder the first 100 bases were extracted for mapping (Additional file 2: Table S1). Post-alignment processing was performed as described in the simulated reads, except that the Mills and 1000G gold standard INDELs were used in local realignment. Variants were identified using the five callers in single-sample calling mode, quality-filtered (at least Q20) and those matching dbSNP v138 were classified as known variants. Given that SNP clusters are prevalent in the HLA region, we did not filter out SNP clusters in the comparison. We compared the called variants to the public call set (see above). The sensitivity, precision rate and overall genotype concordance of known variants were estimated as described in the simulated data.

### Variant calling from chronic lymphocytic leukemia (CLL) exome data

Finally, we selected four of the mappers (exclude NextGenMap that had low sensitivity in the HLA region of NA12878, see the Results section) and three of the callers and applied them to WES data from 22 CLL patients. The selected three callers had relatively high sensitivity in SNP calling (GATK UnifiedGenotyper), INDEL calling (Platypus), or both (GATK HaplotypeCaller) in the HLA region of NA12878 (see the Results section). Buccal cell DNA was collected with written consent from the patients and approval from the institutional review board at Mayo Clinic. Exome capture was carried out using Agilent library capture kit V2 or V4. DNA was sequenced from both ends to 100 bases on a HiSeq2000 machine through the Mayo Clinic Medical Genome Facility.

Mapping, post-alignment processing, variant calling and the classification of variants into known versus novel followed the procedure used for NA12878 WES data. To assess the performance of individual mapper-caller combinations in complex genomic regions, we annotated variants regarding their sequence contexts. Specifically, variants were labeled with ‘LCR’ if they were located in low complexity regions (http://figshare.com/articles/Low_complexity_regions_in_hs37d5/969685) [8] and with ‘SD’ if located in regions of segmental duplications (a minimum of 95 % sequence identity over at least 1 kb, http://humanparalogy.gs.washington.edu/build37/build37.htm). In addition, if three or more SNPs clustered within a window of 20 bp, they were flagged as ‘SnpCluster’.

## Results

### Mapping of simulated reads

We simulated 100-bp paired reads at eight divergence levels (0–15 %) and seven coverage depths (5–100x) from exonic regions of chromosome 6. Five mappers were selected to align the simulated reads to the reference sequence hg19 (Additional file 2: Table S1). The mapping performance was assessed on the 100x coverage datasets in terms of the proportion of accurately mapped reads and that of unmapped reads (Fig. 1; Additional file 1: Figure S1).

At divergence levels of 1 % or lower, the five mappers showed highly comparable mapping accuracy (Fig. 1). At 5–15 % divergence, BWA had over 20 % unmapped rate; of the other four, Stampy and NextGenMap had relative higher mapping accuracy (Fig. 1). On the other hand, Novoalign implements more aggressive soft-clipping than the others, which became most evident at 15 % divergence (Additional file 1: Figure S1).

### Impact of coverage depth on variant calling

Coverage is another key factor in variant calling. We sought to know how coverage might impact different callers at low versus high divergence. A total of 160 cases per coverage depth were evaluated, representing combinations among seven divergence levels, five mappers and five callers, with BWA included at 5–15 % divergence. Not surprisingly, in 93.8 % (150/160) of the cases, the full (100x) coverage datasets showed the highest sensitivity in both SNP and INDEL calling.

**Fig. 2:**
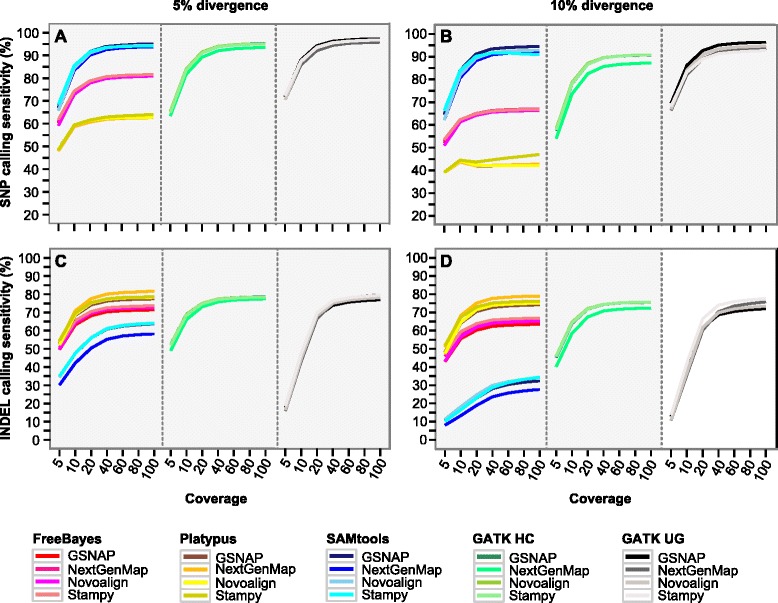
Plotting SNP and INDEL calling sensitivity as a function of coverage depth. *X*-axis indicates coverage depth in simulation and *Y*-axis denotes sensitivity. **a**-**b** SNP calling sensitivity at 5 and 10 % divergence. **c**-**d** INDEL calling sensitivity at 5 and 10 % divergence. The 20 mapper-caller combinations are color-coded, using color gradient to differentiate the four mappers combined with the same caller. *GATK HC* GATK HaplotypeCaller; *GATK UG* GATK UnifiedGenotyper

Profiling sensitivity as a function of coverage should reveal the optimal coverage depth for individual callers; beyond which there would be much less gains in sensitivity. At 1 % or lower divergence, increasing coverage from 5x to 10x led to the biggest increase in sensitivity in both SNP (Additional file 1: Figure S2) and INDEL calling (Additional file 1: Figure S3). In addition, Platypus in SNP calling and GATK UnifiedGenotyper in INDEL calling required a higher coverage depth compared to the others. Overall, at 40x coverage, all callers (except GATK UnifiedGenotyper in INDEL calling) reached 98.4–99.2 % of the SNP calling sensitivity at 100x coverage and 97.4–99.1 % of the INDEL calling sensitivity at 100x coverage, respectively. A similar trend was observed at 5 and 10 % divergence (Figs. 2a-b), where the sensitivity at 40x reached 97.3–100.0 % of that at 100x in SNP calling and 96.5–98.7 % (only 91.1–96.1 % for GATK UnifiedGenotyper) of that in INDEL calling. Therefore, 40x coverage seems sufficient for most of the callers across the full range of divergence.

### Performance of the five variant callers

We tried to identify the callers that would perform well at low and high coverage depths, especially at high divergence. We used 10x to represent low and 40x and 100x to represent high coverage depth, respectively. Since the change of sensitivity from 40x to 100x was very similar among different combinations (Fig. 2; Additional file 1: Figures S2 and S3), the 60x and 80x datasets were not analyzed here. We also excluded the datasets with 15 % divergence since regions with such high divergence should be rare in the genome.

We analyzed each caller by considering all the mappers together, separately at low (0.05–1 %) and high (5–10 %) divergence. In SNP calling, GATK UnifiedGenotyper had the highest sensitivity across all the divergence levels, followed by SAMtools and GATK HaplotypeCaller (Table 1). FreeBayes is known to be less sensitive to highly divergent regions [28]. Indeed, FreeBayes was comparable to the latter two callers at low but was less sensitive (8–28 % lower) at high divergence. Finally, Platypus had the lowest sensitivity in all the datasets. The three highly sensitive callers had similar precision rates (97.8–100.0 %), which were comparable to that of FreeBayes and higher than that of Platypus (Additional file 2: Table S2).

**Table 1:** Percent of SNP and INDEL calling sensitivity in simulated data

Type	Div	Cov	Caller				
	(%)		GATK UG	Platypus	SAMtools	GATK HC	FreeBayes
SNP	0.05–1.00	10	87.3–89.8 (A)	77.6–83.0 (C)	85.9–88.4 (B)	85.4–87.3 (B)	84.1–88.1 (B)
SNP	0.05–1.00	40–100	96.0–98.5 (A)	87.6–96.0 (D)	95.3–98.2 (B)	95.3–97.4 (C)	93.3–98.6 (C)
SNP	5.00–10.00	10	82.2–88.0 (A)	43.7–59.4 (E)	80.7–85.5 (B)	73.6–84.0 (C)	61.3–74.3 (D)
SNP	5.00–10.00	40–100	91.3–97.4 (A)	42.0–64.1 (E)	90.8–95.1 (B)	85.7–95.3 (C)	65.6–81.6 (D)
INDEL	0.05–1.00	10	42.0–53.5 (D)	69.1–74.9 (A)	63.2–73.1 (C)	69.7–74.5 (A)	67.5–75.3 (B)
INDEL	0.05–1.00	40–100	74.2–82.9 (C)	77.8–85.5 (A)	72.6–82.9 (C)	77.9–83.3 (B)	76.0–84.0 (B)
INDEL	5.00–10.00	10	37.4–47.4 (D)	64.1–70.9 (A)	13.3–47.7 (E)	58.4–69.0 (B)	55.5–65.5 (C)
INDEL	5.00–10.00	40–100	70.5–79.7 (B)	72.8–81.7 (A)	23.6–64.1 (D)	70.8–78.6 (B)	62.4–73.7 (C)

Individual datasets are binned into four groups based on coverage (10x or 40–100x) and divergence (0.05–1.00 % or 5–10 %). The values are the range of sensitivity, calculated per caller from the associated mappers and divergence levels. The five callers within each group are ranked (given in parentheses), with “A” indicating the caller with the highest overall sensitivity. *GATK UG* GATK UnifiedGenotyper, *GATK HC* GATK HaplotypeCaller, *Div* divergence, *Cov* coverage

In INDEL calling, overall Platypus and GATK HaplotypeCaller were more sensitive across all the divergence levels. FreeBayes and SAMtools were less sensitive than the above two callers at high divergence (Table 1); GATK UnifiedGenotyper, which requires a higher coverage (Figs. 2c and d), was over 20 % less sensitive than the other callers at 10x coverage (Table 1). In addition, the five callers had roughly similar precision rates (98–100 %) at low divergence, though at 5–10 % divergence Platypus and GATK HaplotypeCaller had reduced precision rates in some cases (Additional file 2: Table S2). Below we analyzed these ideal callers individually to infer the best mapper(s).

### Performance of different mapper-caller combinations

The performance of a caller often varies over alignments generated by different mappers. Thus, we attempted to identify the best mapper(s) for each of the ideal callers identified above, i.e., GATK UnifiedGenotyper, GATK HaplotypeCaller and SAMtools in SNP calling and GATK HaplotypeCaller and Platypus in INDEL calling.

At 0.05–1 % divergence, BWA worked the best in most of the SNP and INDEL calling. The other four mappers also performed well in some cases, particularly at high coverage (Additional file 2: Tables S3 and S4). For example, in SNP calling at 40x or higher coverage, GSNAP and Novoalign performed similarly as BWA for GATK HaplotypeCaller; Stampy had roughly the same sensitivity as BWA for GATK UnifiedGenotyper and SAMtools. In addition, the five mappers had similar precision rates, except that Stampy alignment was about 0.5–2.0 % lower in SNP calling.

For SNP calling at 5–10 % divergence, overall the three callers performed best with GSNAP and with Novoalign and Stampy as well in some cases (Additional file 2: Table S3). The precision rate varied slightly, with GSNAP being 0.4–0.9 % lower than that of Novoalign (data not shown). For INDEL calling at 5–10 % divergence, GATK HaplotypeCaller also achieved similar sensitivities with three of the mappers but had lower sensitivity with NextGenMap (Additional file 2: Table S4). In addition, GATK HaplotypeCaller had roughly the same precision rate over the four mappers; however, its precision rate at 10 % divergence was ~5 % lower than that at 5 % divergence. In contrast, Platypus had the highest sensitivity with NextGenMap (Additional file 2: Table S4). For Platypus, GSNAP alignment had the highest precision rate; however, Stampy alignment was over 6 % lower than the other mappers at 10 % divergence. We ranked the performance of individual combinations based on SNP and INDEL calling sensitivity (Additional file 2: Tables S3 and S4).

Simulated and real exome-seq data are different in several key aspects. The former has a relative uniform distribution of coverage and divergence. However, in the real exome-seq data both features vary widely over different regions, and the types and distribution of variants are much more complicated. Considering the limitations in simulation, below we assessed the same combinations using NA12878 exome-seq data.

### Evaluating SNP calling in NA12878 exome data

The mapping results suggested that replicates 1 and 2 had approximately 62- and 70-fold coverage of the capture regions, respectively, with 85–88 % of the bases having at least 20x coverage. In the assessment, both known (in dbSNP v138) and novel variants were compared to the public call set, where methods that showed higher levels of overlap with known variants in the public call set can be reasonably assumed to have higher sensitivities [9]. The assessment was done mainly on replicate 1, and that on replicate 2 or both replicates was explicitly pointed out. We aimed to sort out the methods that were effective in both highly divergent and typical genomic regions. Toward this, chromosome 6 was split into two entities: the 4-Mb HLA region (29,500,000–33,500,000 bp) with the most extreme divergence and the non-HLA regions representing typical genomic regions.

In the non-HLA regions, 91.3–96.1 % of the SNPs called from Stampy alignments and 96.0–99.4 % from other alignments matched known SNPs. The vast majority of the known SNPs overlapped the public call set (Additional file 1: Figure S4A). Apparently, these methods only varied slightly in the number of known calls (Table 2; Additional file 1: Figure S4A), generally in agreement with the assessment made on the simulation data (1 % or lower divergence and 40–100x coverage, Table 1). On the other hand, the public call set had only 13–15 novel SNPs. However, SNP calling from Stampy alignment had 57–131 novel SNPs, suggesting a reduction of specificity for Stampy. Among the callers, Platypus was enriched in novel SNPs by about 2-fold. Analogous to these observations, we indeed found that Stampy mapping and Platypus calling tend to generate more false positives in simulation data. First, of the five mappers, only Stampy alignment gave rise to false positive calls in the control datasets that have no mismatches with the reference (data not shown). Second, overall Platypus had a slightly lower precision rate in SNP calling, particularly when combined with Stampy (Additional file 2: Table S2).

**Table 2:** Percent of SNP and INDEL calling sensitivity in NA12878

Mapper	Caller	SNP			INDEL	
		Non-HLA	HLA	HLA-DRB1	Non-HLA	HLA
BWA	FreeBayes	95.8	76.0	49.3	72.9	50.0
BWA	GATK HC	94.9	76.9	58.0	83.3	61.3
BWA	GATK UG	96.0	80.5	78.3	74.4	46.7
BWA	Platypus	94.0	71.9	50.7	86.1	60.0
BWA	SAMtools	95.7	75.3	58.2	51.7	48.4
GSNAP	FreeBayes	95.6	80.3	59.4	75.2	46.7
GSNAP	GATK HC	96.1	90.0	72.5	85.7	77.4
GSNAP	GATK UG	97.0	91.7	100.0	76.3	51.6
GSNAP	Platypus	95.2	77.8	42.6	87.7	70.0
GSNAP	SAMtools	96.3	88.3	87.9	55.6	51.6
NextGenMap	FreeBayes	95.5	79.2	48.5	68.6	46.7
NextGenMap	GATK HC	94.1	79.0	62.3	81.5	67.7
NextGenMap	GATK UG	94.5	83.2	76.5	66.9	41.9
NextGenMap	Platypus	92.8	72.8	38.2	76.5	50.0
NextGenMap	SAMtools	94.8	78.6	60.6	47.1	32.3
Novoalign	FreeBayes	94.9	80.1	59.4	76.1	50.0
Novoalign	GATK HC	95.4	89.3	75.4	84.0	67.7
Novoalign	GATK UG	95.6	89.7	85.5	78.2	54.8
Novoalign	Platypus	93.8	79.3	53.6	88.6	73.3
Novoalign	SAMtools	94.7	87.4	83.8	60.2	54.8
Stampy	FreeBayes	96.0	82.7	65.2	81.4	53.3
Stampy	GATK HC	94.9	87.1	72.5	86.4	83.9
Stampy	GATK UG	95.4	87.4	82.6	63.9	51.6
Stampy	Platypus	93.5	78.8	53.7	91.2	73.3
Stampy	SAMtools	95.1	86.6	79.4	68.5	51.7

*HLA* 29,500,000–33,500,000 bp on Chr6; *non-HLA* other capture regions on Chr6; *GATK HC* GATK HaplotypeCaller; *GATK UG* GATK UnifiedGenotyper

**Fig. 3:**
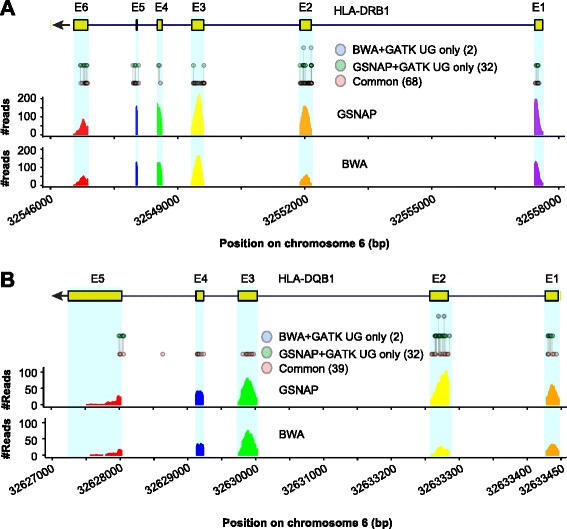
Known SNPs in HLA-DRB1 and HLA-DQB1 from NA12878. The HLA-DRB1 (**a**) and HLA-DQB1 (**b**) structures are shown at the top, with filled boxes representing the exons (E1 to E6 in HLA-DRB1 and E1 to E5 in HLA-DQB1) and arrow indicating transcription direction. The exome-seq reads from NA12878 were mapped by BWA and GSNAP, respectively, and SNPs were called by GATK UnifiedGenotyper (GATK UG). Known SNPs matching dbSNP v138 are showed as ‘circles’ and clustered into three groups. Number in parentheses indicates the number of known SNPs in each group. The two coverage plots depict BWA and GSNAP mapping coverage at base-pair resolution

Compared to the non-HLA regions, there was much larger between-method variability in the HLA region (Figs. 3a and b; Additional file 1: Figure S4B). Sixteen methods had relatively lower SNP calling sensitivity (Table 2), involving BWA or NextGenMap as the mapper or FreeBayes or Platypus as the caller. Of the other nine methods, GSNAP + GATK UnifiedGenotyper was about 2–5 % higher in sensitivity than the others (Table 2). In fact, GSNAP + GATK UnifiedGenotyper covered up to 94.8–99.4 % of the known SNPs identified by each of the other methods.

In HLA-DRB1, the most polymorphic gene out of the five (HLA-A, −B, −C, −DQB1, and -DRB1) in NA12878, the SNP calling sensitivity was strikingly variable, ranging from 38.2 % to 100.0 % (GSNAP + GATK UnifiedGenotyper) (Table 2). For example, GSNAP and BWA together with GATK UnifiedGenotyper identified 102 known SNPs in HLA-DRB1; nevertheless, 32 of them were unique to GSNAP but only 2 unique to BWA (Fig. 3a). Notably, the performance of the five callers on HLA-DRB1 is highly comparable with that on the simulated data (5–10 % divergence and 40–100x coverage, Table 1). A similar pattern was also observed in another highly polymorphic gene, HLA-DQB1 (Fig. 3b). In both genes, exon 2 is excessively divergent, containing >40 % of the known SNPs identified in the capture regions (Figs. 3a and b). However, BWA missed 19 (out of 34) and 12 (out of 41) known SNPs in this exon from HLA-DQB1 and HLA-DRB1, respectively, arguing for the deployment of more sensitive methods.

### Method-specific SNP calls in NA12878

We next examined the known SNPs that were identified by GSNAP + GATK UnifiedGenotyper but missed in the public call set (Table 3), and those that were only present in the public call set (Table 4). GSNAP + GATK UnifiedGenotyper identified 128 unique SNPs from the two replicates (Table 3), with 94 shared between both replicates (29 in HLA-DRB1). By blast search of representative reads spanning the 29 SNPs in HLA-DRB1 against the National Center for Biotechnology Information nucleotide collection (nt) database, we found that 16 of them showed 100 % and another 11 showed 99 % identity with existing HLA-DRB1 sequences.

**Table 3:** GSNAP + GATK UnifiedGenotyper specific SNP calls

Type	Rep	Mapper					No. SNP
		GSNAP	BWA	NextGenMap	Novoalign	Stampy	
Known	1 only	13	3	7	3	4	13 (9,1)
Known	2 only	21	2	8	5	4	21 (10,0)
Known	1 & 2	94	32	70	60	51	94 (75,0)
Novel	1 only	10	1	1	1	3	10 (5,3)
Novel	2 only	11	5	3	4	4	11 (2,0)
Novel	1 & 2	18	5	10	8	11	18 (12,1)

Shown is the number of SNPs in the HLA region of NA12878 that were called by GSNAP + GATK UnifiedGenotyper but missed in the public call set. The GSNAP + GATK UnifiedGenotyper specific SNPs included those only identified from replicate 1 (“1 only”), from replicate 2 (“2 only”), and from both (“1 & 2”). The number of SNPs identified by GATK UnifiedGenotyper together with the other four mappers was also shown here. The last column is the total number of SNPs, and those in SNP cluster and segmental duplications were shown in parentheses. *Known* SNP matching dbSNP v138; *novel* SNP not matching dbSNP v138, *Rep* replicate; *HLA* Chr6:29,500,000–33,500,000 bp

**Table 4:** Public call set specific SNPs

Type	Rep	Public call set				No. SNP
		Cortex	DISCOVAR	GATK HC	Conf	
Known	1 only	6	13	17	2	21 (11,0)
Known	2 only	3	5	11	4	11 (3,2)
Known	1 & 2	4	18	48	6	57 (33,10)
Novel	1 & 2	1	10	4	0	13 (2,5)

Shown is the number of SNPs in the HLA region of NA12878 that were present in the public call set but missed by GSNAP + GATK UnifiedGenotyper. Cortex, DISCOVAR and GATK HaplotypeCaller (GATK HC) calls were from 250-bp paired sequencing of a PCR-free genomic library [38]. The high-confident call set (“Conf”) was from [36]. See Table 3 footnote for additional information

The SNPs unique to GSNAP + GATK UnifiedGenotyper were enriched in SNP cluster. About three-fourths (94/128) were in SNP cluster, compared to only 2 % of the total SNPs in the non-HLA regions and 38 % in the HLA region. Misalignments around INDELs (within 10-bp) can lead to high false positives [9, 26]. However, 85.2 % (109/128) of the unique SNPs was at least 50 bp away from known INDELs, ruling out misalignments as being a major source of these unique calls. Also, 64.1 % (82/128) was identified by at least another two mappers with GATK UnifiedGenotyper. Finally, we manually checked GSNAP alignments in regions surrounding the 94 SNPs shared by both replicates. Two of them were low-confident calls in both replicates (two out of four to five supporting reads were soft-clipped) and another two were each supported by only two reads (out of two in total) in replicate 1; all the others were fully supported by the alignments. We reason that the vast majority of the known SNPs called by GSNAP + GATK UnifiedGenotyper but missed in the public call set represent true variants.

On the other hand, in GATK UnifiedGenotyper calling, GSNAP missed a total of 89 known SNPs present in the public call set (Table 4), including 66 missed by all the five and 13 by four of the mappers (except Stampy that had reduced specificity in simulated data). Over half of the missed SNPs were in SNP cluster. Of the five HLA genes, only HLA-DQB1 showed an obvious loss of SNP calls (12 SNPs, see below for manual inspection). We traced the 89 public call set specific SNPs back to the methods identifying them. While all of them were identified from the 250-bp genomic sequencing data [38], only 12 were in the high-confident call set [36].

We manually checked GSNAP alignments for reads spanning the 57 SNPs that GATK UnifiedGenotyper failed to call in both replicates. Two of them appeared to be false negatives, with one overlapping a 3-bp INDEL in HLA-DQB1. Twenty-eight miscalls were due to extremely low coverage (0–2 reads) or insufficient supporting reads (0–2 out of 9–29 mapped reads). Another 11 SNPs were at sites with high coverage (87–226x) in both replicates, but only 2–10 % of the reads supported the alternative calls. Of the remaining 16 SNPs (57–2–28–11 = 16), five had 31–75x and 11 had 142–178x coverage; nevertheless, only 0–4 reads supported the SNPs, raising the possibility that they are platform-specific calls or simply false positives in the public call set. Twelve of the 57 missed SNPs were in HLA-DQB1; 11 of them had two or fewer supporting reads. We argue that a significant proportion of the known SNPs unique to the public call set is likely identifiable only through whole genomic sequencing, longer reads and/or by *de novo* assembly-based calling methods. Collectively, these results strongly support the findings from the simulated data (5–10 % divergence, Table 1; Additional file 2: Table S3).

With GATK UnifiedGenotyper, GSNAP identified 39 novel SNPs that were not in the public call set (Table 3) but missed 13 in the public call set (Table 4). Fifteen of the 39 SNPs were identified by at least three mappers, supporting the reliability of these calls. However, none of the 13 SNPs were detected by any of the mappers; they were all absent from the high-confident call set [36]. Thus, these public specific novel SNPs more likely represent platform-specific calls.

### Evaluating INDEL calling in NA12878 exome data

As expected, it is more difficult to detect INDELs from the HLA regions than from the non-HLA region (Table 2). GATK HaplotypeCaller and Platypus [31] implement local *de novo* assembly, a feature that should contribute to INDEL detection. Indeed, the two callers had higher sensitivity in both HLA and non-HLA regions (Table 2; Additional file 1: Figures S4C and S4D; Additional file 2: Table S5). In the non-HLA regions, FreeBayes and GATK UnifiedGenotyper had similar sensitivities, with SAMtools being least sensitive (Table 2); in the HLA region, these three callers had comparable sensitivity. The results largely agree with the findings from simulated data summarized in Table 1.

As for the novel INDELs, we focused on those that were identified by four of the mappers (exclude NextGenMap that had the lowest sensitivity) in combination with Platypus and GATK HaplotypeCaller. Overall, the number of public call set- (26–31) and method-specific INDELs (22–44) was not markedly different in the non-HLA regions. In the HLA region, however, there were two to ten times more novel INDELs unique to the public call set than unique to each of the methods (Additional file 2: Table S6). GATK HaplotypeCaller identified much more novel INDELs than Platypus in the HLA region. About 80 % of the GATK HaplotypeCaller novel calls were supported by at least two mappers and also overlapped the public call set, supporting their authenticity.

### Variant calling at different mapping parameter settings in NA12878

**Fig. 4:**
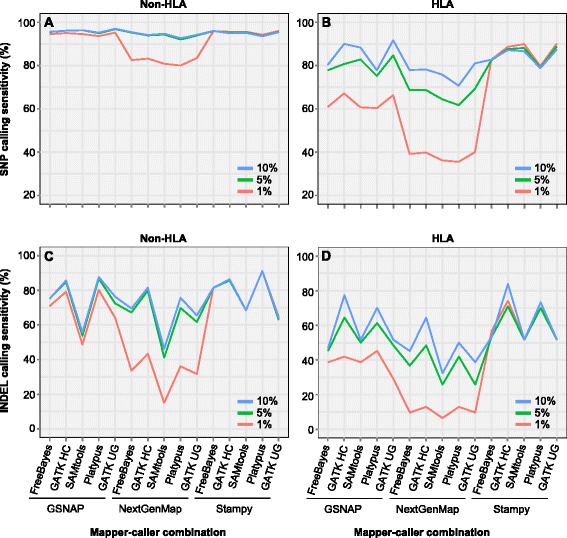
Variant calling sensitivity in NA12878 at three divergence settings. **a** SNP calling in non-HLA regions. **b** SNP calling in HLA region. **c** INDEL calling in non-HLA regions. **d** INDEL calling in HLA region. The three divergence levels in mapper parameter settings are 1, 5 and 10 %. Reads were aligned to the hg19 reference sequence by the three mappers at each of the divergence settings. *GATK HC* GATK HaplotypeCaller; *GATK UG* GATK UnifiedGenotyper

Divergence level between reads and the reference genome is a critical factor in variant discovery. Of the five mappers, GSNAP, NextGenMap and Stampy provide the parameter to specify the level of divergence (or identity). To understand how this parameter impacts variant calling efficiency, we tested three different divergence levels, 1, 5 and 10 % (GSNAP v2013–10–25 allows no more than 10 % divergence) for these three mappers. In the non-HLA regions, SNP calling from NextGenMap alignments at the divergence settings of 5 % and 10 % was 11–14 % higher in sensitivity compared to that from NextGenMap using 1 % divergence (Fig. 4a); in the HLA region, the differences in sensitivity were increased by about 3-fold (Fig. 4b). For GSNAP, the SNP calling sensitivity was highly comparable across the three settings in the non-HLA regions; while in the HLA region, GSNAP alignments at the setting of 10 % divergence showed the highest sensitivity (Figs. 4a and b). Finally, for Stampy, nearly no difference was observed in both HLA and non-HLA regions (Figs. 4a and b). Therefore, for GSNAP, which overall performs the best in terms of SNP calling sensitivity and specificity, the 10 % divergence we used throughout the analysis represents the ideal setting. The same trend was observed in INDEL detection from non-HLA and HLA regions (Figs. 4c and d), where the highest sensitivity was achieved with the parameter of 10 % divergence for both GSNAP and NextGenMap.

### Variant discovery from CLL exome-seq data

Finally, in order to ascertain the broad application of the selected variant calling methods (see below), we generated 100-bp WES data from a cohort of 22 CLL patient samples. Reads mapping (exclude NextGenMap due to its low sensitivity) and variant calling followed the procedure used in NA12878. The average per-base coverage varied between 67x and 113x, with 82.4–94.9 % of the bases having at least 20x coverage. As demonstrated in both simulated (Table 1) and NA12878 WES data (Table 2), GATK UnifiedGenotyper and Platypus are effective for SNP and INDEL calling, respectively, while GATK HaplotypeCaller are ideal for both. Focusing on known variants, we asked whether these three callers also outperformed the others on the CLL WES data, particularly in the HLA region that contains CLL susceptibility loci [40, 41].

Indeed, in the non-HLA regions, the number of known SNPs per sample differed by 35–123 (2.4–8.8 % of the total) among the 12 methods (Additional file 1: Figure S5A; Additional file 2: Table S7). Overall GATK UnifiedGenotyper with Stampy, GSNAP and BWA identified slightly more known SNPs. In contrast, there were much larger differences (146–467 known SNPs or 19.3–70.3 % of the total known) in the HLA region, with GSNAP + GATK UnifiedGenotyper detecting the most and BWA + Platypus detecting the least or near the least number of known SNPs (Additional file 1: Figure S5B; Additional file 2: Table S7). Of the five highly polymorphic HLA genes, GSNAP + GATK UnifiedGenotyper performed the best in HLA-A, −B, −C and -DRB1, and equally well with Stampy + GATK UnifiedGenotyper in HLA-DQB1 (Additional file 1: Figures S6A-S6E). GATK UnifiedGenotyper, which had a high specificity (Table 3; Additional file 2: Table S2), showed the highest sensitivity, followed by GATK HaplotypeCaller and Platypus, consistent with the inference made from both simulated (Table 1) and NA12878 data (Table 2).

For INDEL calling in the non-HLA regions, Platypus was more sensitive than GATK HaplotypeCaller and GATK UnifiedGenotyper (Additional file 2: Table S8). Four of the mappers made nearly no difference, with the exception of Stampy that identified about 10 % less INDELs in GATK UnifiedGenotyper calling. Reversely in the HLA region, with BWA excluded, GATK HaplotypeCaller identified more known INDELs than the other two callers (Additional file 2: Table S8). Together with GATK HaplotypeCaller, GSNAP identified the most known INDELs in the HLA region from 19 of the 22 samples, followed by Novoalign and Stampy. GATK HaplotypeCaller was also more sensitive to novel INDELs (Additional file 2: Table S8), as revealed in NA12878 (Additional file 2: Table S6).

In summary, for SNP calling, GATK UnifiedGenotyper is generally more powerful than GATK HaplotypeCaller in the HLA region, especially with GSNAP; in the non-HLA regions, these two callers are roughly comparable and both are better than Platypus. For INDEL calling, GATK HaplotypeCaller and Platypus are the two best callers. In the non-HLA region, Platypus is superior to GATK HaplotypeCaller; while in the HLA region, GATK HaplotypeCaller is often better than Platypus. GSNAP and Novoalign are ideal mappers for both SNP and INDEL calling. One limitation with GSNAP (also Novoalign) is that it is over four times slower than BWA (data not shown). A possible solution would be to first map reads using BWA, extract pairs with unmapped read(s), and then re-map those using GSNAP. We tested its feasibility on the NA12878 WES data. Remarkably, in GATK UnifiedGenotyper and HaplotypeCaller calling, this two-step mapping approach recovered >99.5 % of the known SNPs previously identified from chromosome 6 using GSNAP alone. On the other hand, Stampy is oversensitive, which results in more false positive calls.

**Fig. 5:**
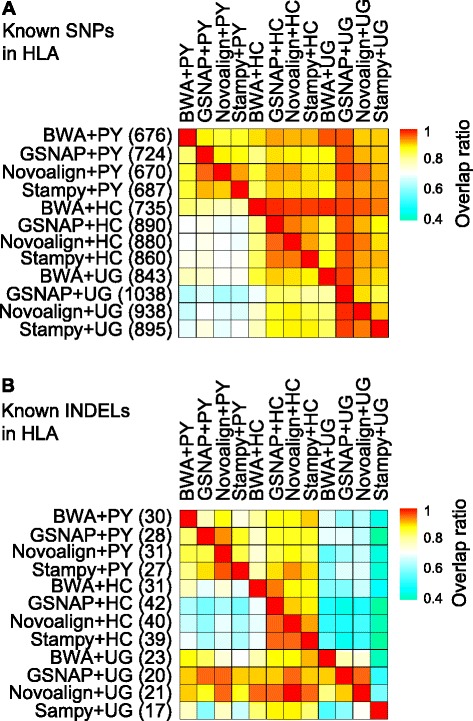
Overlap of known variants in the HLA region of the CLL sample 612703. **a** Overlap of known SNPs. **b** Overlap of known INDELs. The 12 call sets were generated by three callers together with four mappers. Number of known variants is shown in parentheses. Each non-triangle box is pseudo-colored to signify the proportion of the call set on the left that is overlapped by the call set showed on the top. *HC* GATK HaplotypeCaller; *PY* Platypus; *UG* GATK UnifiedGenotyper

We next focused on one of the five CLL samples (ID 612703) with an average per-base coverage exceeding 100x (103–105x, depending on the mapper used), using four mappers (exclude NextGenMap) and three callers (exclude SAMtools and FreeBayes). For known SNPs, the 12 methods showed 95–99 % overlap between each other in the non-HLA regions (Additional file 1: Figure S7A), versus 62–98 % in the HLA region (Fig. 5a). As observed in NA12878 (Table 2), GATK UnifiedGenotyper performed better than GATK HaplotypeCaller and Platypus in the HLA region, with GSNAP + GATK UnifiedGenotyper being most sensitive (Fig. 5a; Additional file 1: Figures S7B and S7C). It covered over 90 % of the known SNPs identified by five of the methods together in HLA and HLA-DRB1 (Additional file 1: Figures S8A and S8B).

For INDEL discovery, Platypus performed the best in the non-HLA regions (Additional file 1: Figure S9) and GATK HaplotypeCaller in the HLA region (Fig. 5b), as previously revealed across the CLL cohort (Additional file 2: Table S8). GATK HaplotypeCaller together with Novoalign or GSNAP was ideal for INDEL detection in the HLA region, Platypus and GATK HaplotypeCaller together allowed more complete INDEL discovery in both non-HLA and HLA regions (Additional file 1: Figures S10A and S10B).

## Discussion

Accurate variant discovery is crucial for pinpointing the causal mutations underlying human diseases. Current computational methods are generally effective in detecting ordinary variants but less so for variants located in difficult regions [38]. One of those regions is the HLA region, which is clinically important but extremely divergent. Focusing on chromosome 6 we comprehensively assessed five popular mappers together with five callers on both simulated and real WES data from NA12878. We have developed an analytical workflow that allows more accurate variant discovery in the HLA region and across the genome.

Our analysis revealed marked difference among the five callers at high divergence. GATK UnifiedGenotyper performed the best in single-sample SNP calling, especially with GSNAP, on simulated data at 5–10 % divergence. All but Platypus had similarly high precision rates. GATK UnifiedGenotyper was also about 1–6 % higher in sensitivity than the other callers in vast majority of the cases with low divergence. For INDEL calling in simulated high divergence data, GATK HaplotypeCaller and Platypus were generally more sensitive, but at the cost of reduced specificity. We revealed a similar trend of performance for these methods in the HLA and non-HLA regions in NA12878. In summary, GATK UnifiedGenotyper, SAMtools and GATK HaplotypeCaller are ideal for SNP calling while GATK HaplotypeCaller and Platypus are more effective for INDEL calling.

The mapping accuracy is often calculated by considering only the alignment start position, which does not always reflect the true alignment status of individual bases. In addition, optimal pairwise alignments between individually mapped reads and the reference sequence may not guarantee high confidence in multiple alignments. Therefore, different mappers and callers need to be assessed together in order to identify the best combination(s). The five mappers are known to vary in mapping highly divergent reads [12–14], which we also revealed in our simulated data. Even with a similar mapping rate, two mappers can perform quite differently in the context of variant calling. For example, GSNAP [14] and NextGenMap [13] are both designed to map highly divergent reads and we observed roughly comparable mapping accuracy at 5–10 % divergence. However, for the three sensitive callers in SNP detection, NextGenMap is obviously less suitable than GSNAP, evident by a loss of 8.5–11.0 % sensitivity in the HLA region of NA12878. To further support this, we found that, of the five GSNAP-mapped read pairs that contained six known SNPs in a 50-bp region within HLA-DRB5 (32,489,626–32,489,675 bp), NextGenMap mapped only one of the ends correctly (to this 50-bp region) but the other end to regions of 36–63 kb away. On the other hand, there is also obvious difference across different callers given the same mapper. Using GSNAP as the mapper, GATK UnifiedGenotyper was 2.0–4.4 % more sensitive than GATK HaplotypeCaller and SAMtools and 12–14 % more sensitive than the other two in SNP calling from the HLA region. We analyzed two additional NA12878 WES datasets generated using Illumina Nextera Rapid Capture Exomes capture kit (SRR1919605) and Roche Life Science SeqCap EZ Human Exome Library v3.0 (SRR1611181). GSNAP + GATK UnifiedGenotyper also showed the highest sensitivity in SNP calling in the HLA region (data not shown). Our analysis has identified GSNAP + GATK UnifiedGenotyper as the most sensitive method for SNP detection in both HLA and non-HLA regions.

Strikingly, GSNAP + GATK UnifiedGenotyper achieved 100 % sensitivity in HLA-DRB1, HLA-A and HLA-C, three highly polymorphic genes in NA12878. In HLA-DRB1, for example, this method identified all the 70 known SNPs annotated in the public call set [36, 38], plus an additional 30 unique known calls. In contrast, the widely used BWA + GATK UnifiedGenotyper missed >30 % of the known SNPs in this gene. Comparable results were obtained for GATK HaplotypeCaller and Platypus in INDEL calling.

Besides the HLA region, there are many other regions in the human genome that are also highly divergent [20]. For example, the 1000 Genomes Project identified large regions on chromosomes 8 (about 15 Mb) [42] and 16 and subtelomeric regions on autosomal chromosomes that have high SNP density [43]. We have identified a few ideal mapper-caller combinations that are sensitive to both highly divergent regions and regions with low mutation rates, such as GSNAP + GATK UnifiedGenotyper in SNP calling and GSNAP + GATK HaplotypeCaller and GSNAP + Platypus in INDEL calling. For INDEL calling, GSNAP + GATK HaplotypeCaller is more sensitive to the HLA region while GSNAP+ Platypus is more sensitive to the non-HLA regions. A joint calling with both methods should be the appropriate approach for genome-wide INDEL detection.

Traditionally, genotyping in the HLA region often relies on microarray hybridization [24] or sequencing PCR amplicons that targeted selected exons [23, 44] or entire genes [45], which are costly, time-consuming and low throughput. In addition, current studies often focus on known variants in the international ImMunoGeneTics project (IMGT)/HLA Database without considering novel variants [46, 47]. Lastly, though WES was used for HLA genotyping and variant discovery, the standard mapping-based approaches did not work well [48]. Our approach represents a more generalized methodology, which is effective for genome-wide variant detection but particularly sensitive in highly divergent regions like HLA. Though only tested on WES, it should be applicable to whole genome sequencing data as well.

## Conclusions

We aimed to develop a strategy enabling more accurate variant discovery in highly divergent regions. Focusing on the HLA region that shows extreme divergence across different haplotypes, we revealed marked differences among the methods in SNP and INDEL calling from NA12878 WES data. We captured a similar trend on WES data from a cohort of CLL patients. Specifically, GSNAP and Novoalign achieve high sensitivity in mapping divergent reads without losing the specificity. Their limitation in speed could be overcome through a two-step mapping approach, in which reads are first mapped by BWA and unmapped ones are then re-mapped by GSNAP or Novoalign. Together with these two mappers, GATK UnifiedGenotyper demonstrates its excellence in SNP calling, followed by GATK HaplotypeCaller and SAMtools; in INDEL calling, GATK HaplotypeCaller and Platypus outperform the others and their joint calling clearly enhances the outcome. Given that highly polymorphic regions are distributed over many chromosomes and are often associated with human disease, our study brings additional options into the current variant calling practice.

## Additional files

Additional file 1: Figure S1. Accumulative mapping rate of four aligners. **Figure S2.** SNP calling sensitivity at 0.05–1 % divergence. **Figure S3.** INDEL calling sensitivity at 0.05–1 % divergence. **Figure S4.** Number of known variants in HLA and non-HLA regions of Chr6 in NA12878. **Figure S5.** Number of known SNPs in the HLA and non-HLA regions of Chr6 in CLL. **Figure S6.** Number of known SNPs in five HLA genes. **Figure S7.** Heat maps illustrating the overlap of known SNPs. **Figure S8.** Venn diagrams depicting the overlap of known SNPs. **Figure S9.** Overlap of known INDELs in the non-HLA regions of Chr6. **Figure S10.** Venn diagrams depicting the overlap of known INDELs. (PDF 2895 kb) Additional file 2: Table S1. Five mappers and five variant callers used in the study. **Table S2.** SNP and INDEL calling precision rate in simulated data. **Table S3.** Percent of SNP calling sensitivity for three callers in simulated data. **Table S4.** INDEL calling sensitivity for two callers in simulated data. **Table S5.** Known INDELs in the HLA region of NA12878. **Table S6.** Novel INDELs in the HLA region of NA12878. **Table S7.** Number of novel SNPs in 22 CLL samples. **Table S8.** Number of INDELs in 22 CLL samples. (PDF 170 kb)

## Abbreviations

Bp: Base pair
BWA: Burrows-Wheeler aligner
Chr6: Chromosome 6
CLL: Chronic lymphocytic leukemia
GATK: Genome Analysis Toolkit
HLA: Human leukocyte antigen
INDEL: Insertion and deletion
PCR: Polymerase chain reaction
SNP: Single nucleotide polymorphism
WES: Whole exome sequencing
