# Supplementary material for: An analytical workflow for accurate variant discovery in highly divergent regions
Source: BMC Genomics. 2016 Sep 2;17(1):703. doi: 10.1186/s12864-016-3045-z (PMC5010666; doi:10.1186/s12864-016-3045-z)
Supplement: Additional file 1: Figure S1. — Accumulative mapping rate of four aligners. Figure S2. SNP calling sensitivity at 0.05–1 % divergence. Figure S3. INDEL calling sensitivity at 0.05–1 % divergence. Figure S4. Number of known variants in HLA and non-HLA regions of Chr6 in NA12878. Figure S5. Number of known SNPs in the HLA and non-HLA regions of Chr6 in CLL. Figure S6. Number of known SNPs in five HLA genes. Figure S7. Heat maps illustrating the overlap of known SNPs. Figure S8. Venn diagrams depicting the overlap of known SNPs. Figure S9. Overlap of known INDELs in the non-HLA regions of Chr6. Figure S10. Venn diagrams depicting the overlap of known INDELs. (PDF 2895 kb) [file 12864_2016_3045_MOESM1_ESM.pdf]

Additional file 1

Supplementary methods

1. Simulating exome-seq reads from chromosome 6

To identify regions from chromosome 6 (Chr6) for simulation, we used exons from hg19 refGene annotation (as of 12/10/2013) together with capture regions included in any of the four Agilent SureSelectXT Human All Exon kits: All Exon 50Mbp, All Exon V4, All Exon V4+UTRs and All Exon V5+UTRs (http://www.agilent.com). To ensure full coverage for short exons and exon edges, the refGene exons were each extended by +/-100 bp and the extended coordinates were retrieved from the UCSC table browser (http://genome.ucsc.edu/cgi-bin/hgTables). The four All Exon kits each had between 121 and 406 kb unique sequences from Chr6 that were not covered by the coordinate-extended exons. Therefore, we merged the above five lists to generate 10,768 non-overlapping regions, with a median size of 350 bp (range: 89-15,378 bp), totaling 6.4 Mbp.

Simulation was done using Dwgsim, a whole genome simulation tool (https://github.com/nh13/DWGSIM/wiki). Dwgsim was run separately at each of the seven divergence levels (between 0.05% and 15%) and for the control, using the same parameter settings: 90% SNPs, 10% INDELs, inner distance of 200 bp, no random DNA read, and random seed of 123. We did not specify error rate in Dwgsim simulation (options –e and –E). To mimic the non-random distribution of base quality in real sequencing reads, we assigned empirical per-base quality scores to simulated reads by replacing the quality-score lines in the FASTQ files with those randomly taken from our CLL exome-seq data. Dwgsim outputs a VCF file, which shows the Chr6 positions, strand information and genotypes of all the permuted (preplaced) SNPs and INDELs, and a FASTQ file with 2.43 million pairs of simulated reads.

2. Evaluating mapping accurate for simulated reads

BWA is effective in mapping reads with relatively low divergence from the reference (<2%) [1]. The other four mappers are capable of aligning reads to more divergent regions, including GSNAP [2], NextGenMap [3], Novoalign (http://www.novocraft.com/), and Stampy [4]. Each mapper was run with the parameter settings provided in Additional file 2: Table S1. The alignment in the sequence alignment map (SAM) format was converted into the binary alignment map (BAM) format using SAMtools [5] and sorted by coordinates using Picard SortSam command (http://picard.sourceforge.net/).

We assessed the five mappers based on mapping rate and accuracy. Four of the mappers (except Stampy) apply 'soft-clipping', which masks 5' and 3' unalignable termini of a read and report the chromosome position at which the actual alignment starts. As some of the simulated variants can be located at 5' and/or 3' in reads, soft-clipping will cause discrepancy in coordinates even when a read is indeed mapped back to its original location. Therefore, for each mapped read, we estimated the mapping position by adding the soft-clipped bases to the reported mapping position. The number of soft-clipped bases was extracted from the CIGAR string in the BAM file.

3. Evaluating variant calling from simulated data

Prior to variant calling, we processed the alignments following the published procedure [6] but without base quality score recalibration. The base quality score recalibration step is expected to improve variant calling by providing more accurate base quality scores. We skipped this step, since we found that it reduced the variant calling sensitivity in highly divergent regions (Tian *et al.*, unpublished data). Duplicates in the coordinates-sorted BAM files were marked by the Picard MarkDuplicates command. As bases spanning INDELs have a high chance of being incorrectly aligned to the reference, we used GATK IndelRealigner command to perform local realignment around 90%, rather than all of the preplaced (permuted) INDELs. This selection is based on the notion that, in variant calling from real exome-seq data, local realignment is performed

around known INDELs, which are a subset of all the INDELs in a sample. Processed alignments were then used in variant calling by GATK UnifiedGenotyper and HaplotypeCaller, FreeBayes, SAMtools mpileup and Platypus. Variants (SNPs and short INDELs) were identified from the regions used in simulation. Among these callers, Platypus and FreeBayes report a single event for multiple SNPs within a stretch of 5 bp or shorter, called multiple-nucleotide polymorphism (MNP) [7]. To increase the comparability among different callers, multiple-nucleotide polymorphisms were decomposed into individual events using GATK walker VariantsToAllelicPrimitives.

We evaluated variant calling from simulated data on the basis of sensitivity, precision rate, and overall genotype concordance reported by the GATK walker GenotypeConcordance. For INDELs of lengths of 2 bp or longer, all callers report the most left position based on left-normalization. Considering this, in estimating levels of overlap the original positions of permuted INDELs were extended by +/- 5 bp before intersecting with the positions of called INDELs using BEDTools [8].

Sensitivity is estimated using the formula:
Sensitivity = true positive/(true positive + false negative)

Where true positive refers to the number of true variants identified by a caller, while false negative represents the number of true variants missed by a caller. Here, the true variants are preplaced (simulated) ones reported in the dwgsim output VCF file.

Precision rate is estimated as:
Precision rate = true positive/(true positive + false positive)

Where false positive represents the number of called variants that does not overlap the true (simulated) variants.

4. Calling variants from public exome-seq data in NA12878

We downloaded two 150-base paired-end exome-seq data sequenced by the Illumina, Inc. in NA12878 (https://basespace.illumina.com/analyses/6847907/inputs). The two replicates (FC1_NA12878_01 and FC1_NA12878_04) each has ~100x coverage on average. The first 100 bases were extracted and mapped by the five aligners. As done for the simulated reads, the mapping output SAM files were converted into BAMs, position sorted and duplicate marked. Local realignment was then performed around known INDELs, i.e., the Mills and 1000G gold standard INDELs (Mills_and_1000G_gold_standard.indels.hg19.vcf.gz) included in the GATK resource bundle for hg19.

Variants were identified using the five callers, and only those from the capture regions on Chr6 that have a phred-scaled quality score of at least 20 were retained. They were split into 'known' and 'novel' by intersecting with dbSNP v138 (ftp://ftp.broadinstitute.org/bundle/2.8/hg19/dbsnp_138.hg19.vcf.gz), requiring exact match in genomic position for an INDEL to be classified as 'known'. By treating the union of the four public call sets below as the 'true' variants in NA12878, the sensitivity, precision rate and overall genotype concordance of known SNPs and INDELs were estimated, as described in the simulated data. The first set was called using GATK UnifiedGenotyper, GATK HaplotypeCaller and Cortex from 11 whole genome and three exome sequencing datasets, available at ftp://ftp-trace.ncbi.nih.gov/giab/ftp/data/NA12878/variant_calls/GIAB_integration/NIST_RTG_PlatGen_merged_highconfidence_v0.2.primitives.vcf.gz [9]. The other three were generated by three packages, Cortex, GATK HaplotypeCaller and DISCOVAR, from 250-bp read pairs (http://genepattern.broadinstitute.org/ftp/distribution/crd/DiscovarManuscript/vcf/) [10].

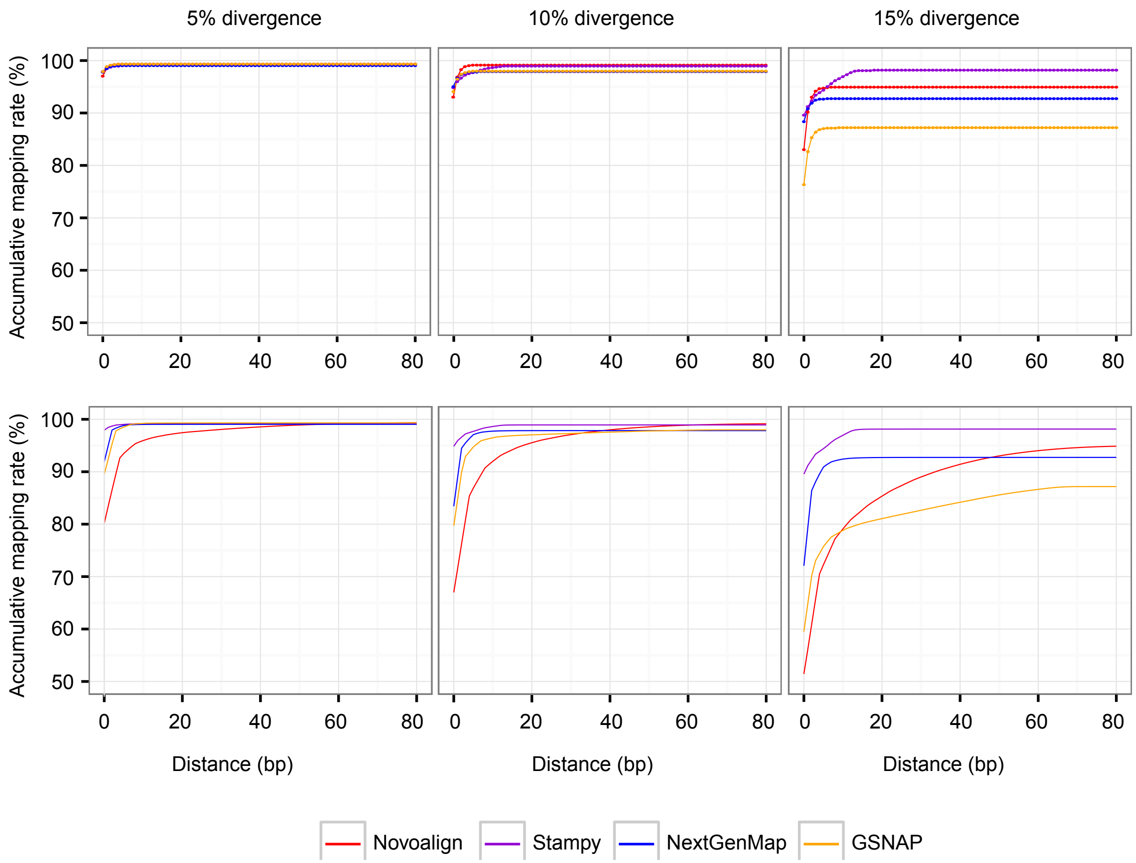

Figure S1. Accumulative mapping rate of four aligners. Accumulative mapping rate (*y*-axis) is plotted as a function of the distance (bp, *x*-axis) from the original position to the mapping position on chromosome 6. Upper panels: the number of soft-clipped bases was estimated from the CIGAR string in the BAM file and added back to the reported mapping position to generate the final mapping position; lower panels: the soft-clipped bases were not added back. Only the 100x simulation datasets with 5-15% divergence were used. BWA has poor performance at high divergence and is not shown here.

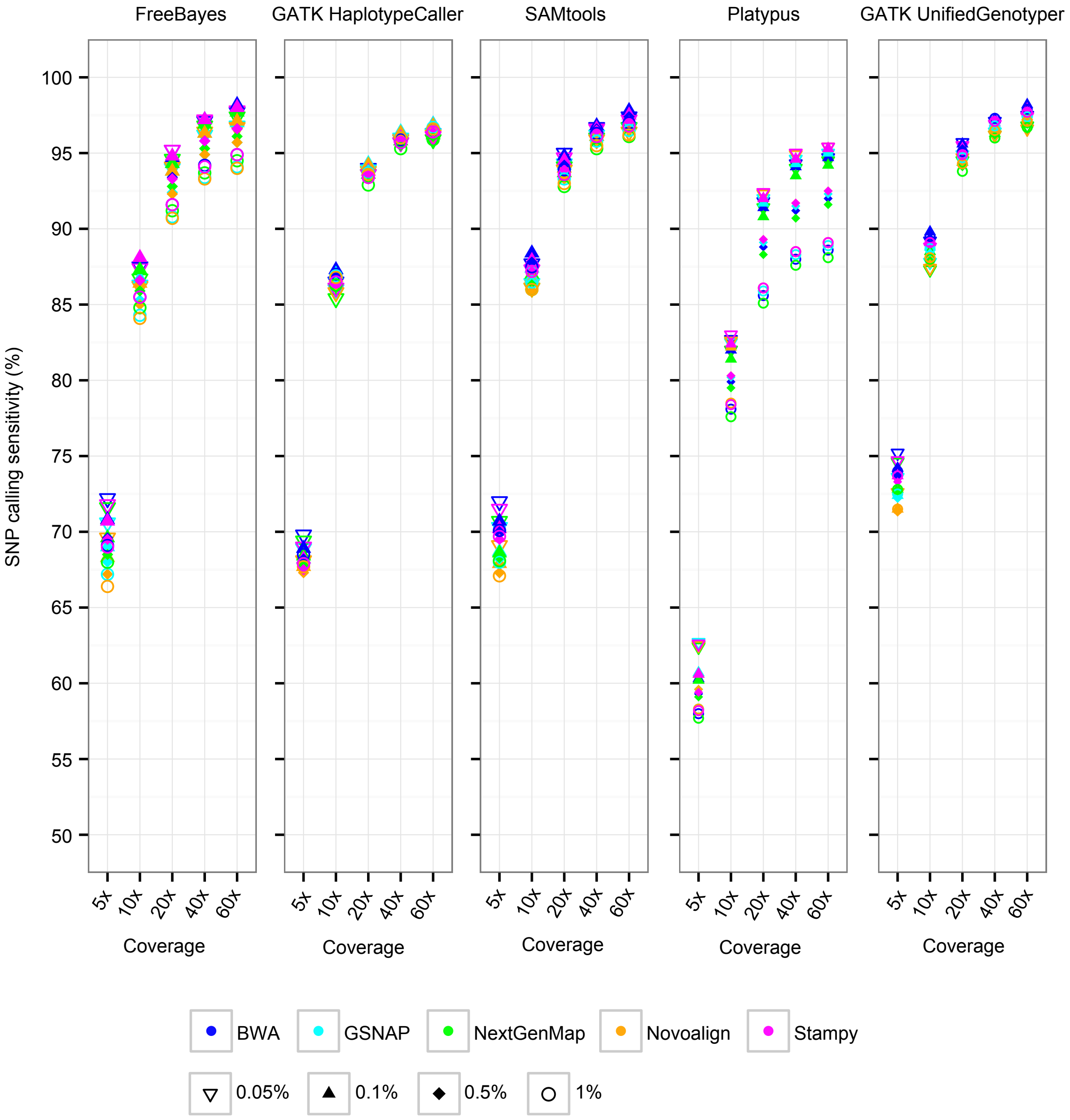

Figure S2. SNP calling sensitivity at 0.05-1% divergence. Sensitivity is calculated after local realignment**.** For each caller at each coverage depth, sensitivities from the associated five mappers (by color) and four divergence levels (0.05-1%, by shape) were plotted together. Only coverage depths (*x*-axis) from 5x to 60x are displayed.

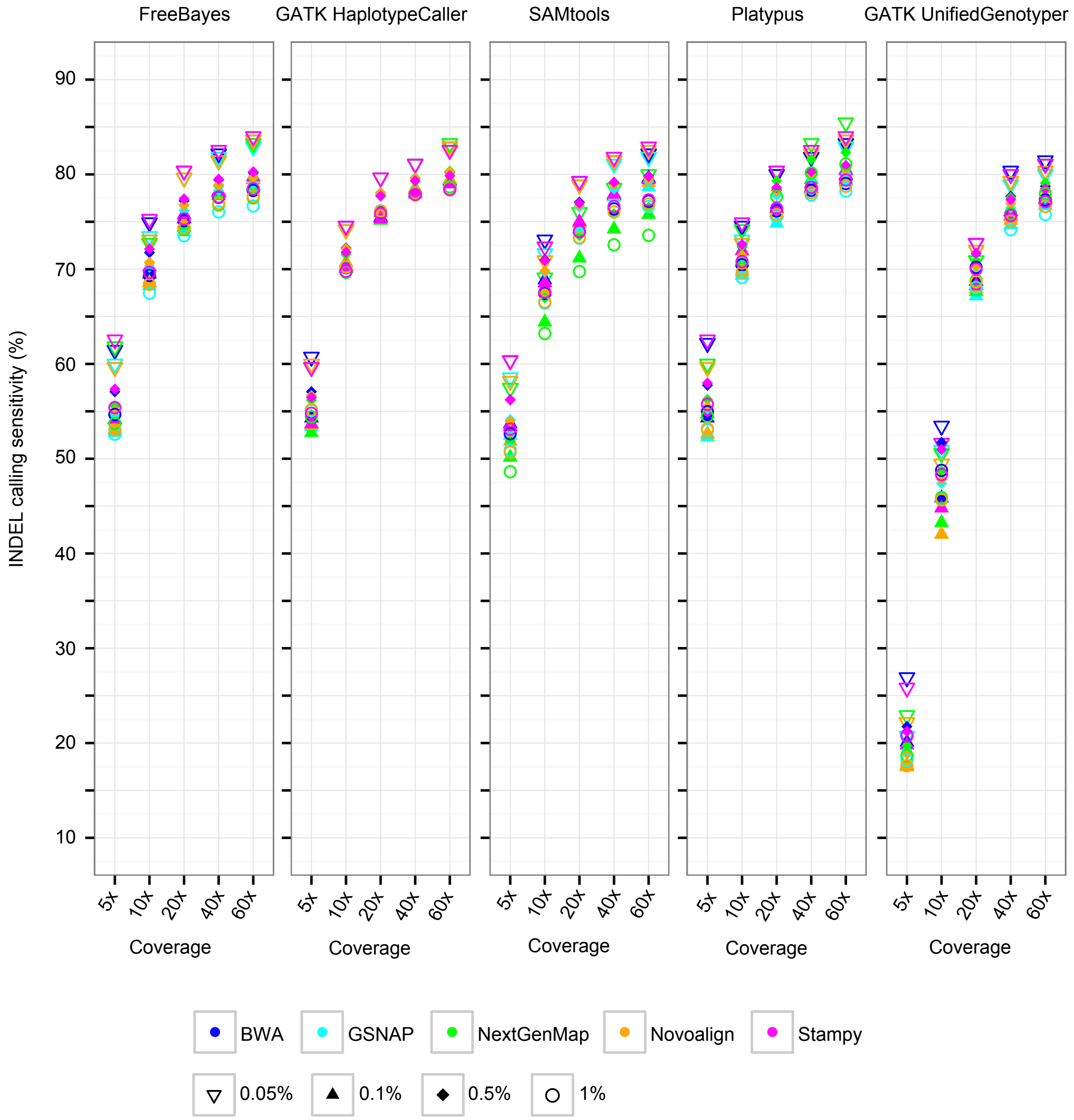

Figure S3. INDEL calling sensitivity at 0.05-1% divergence. See Figure S2 legend for details.

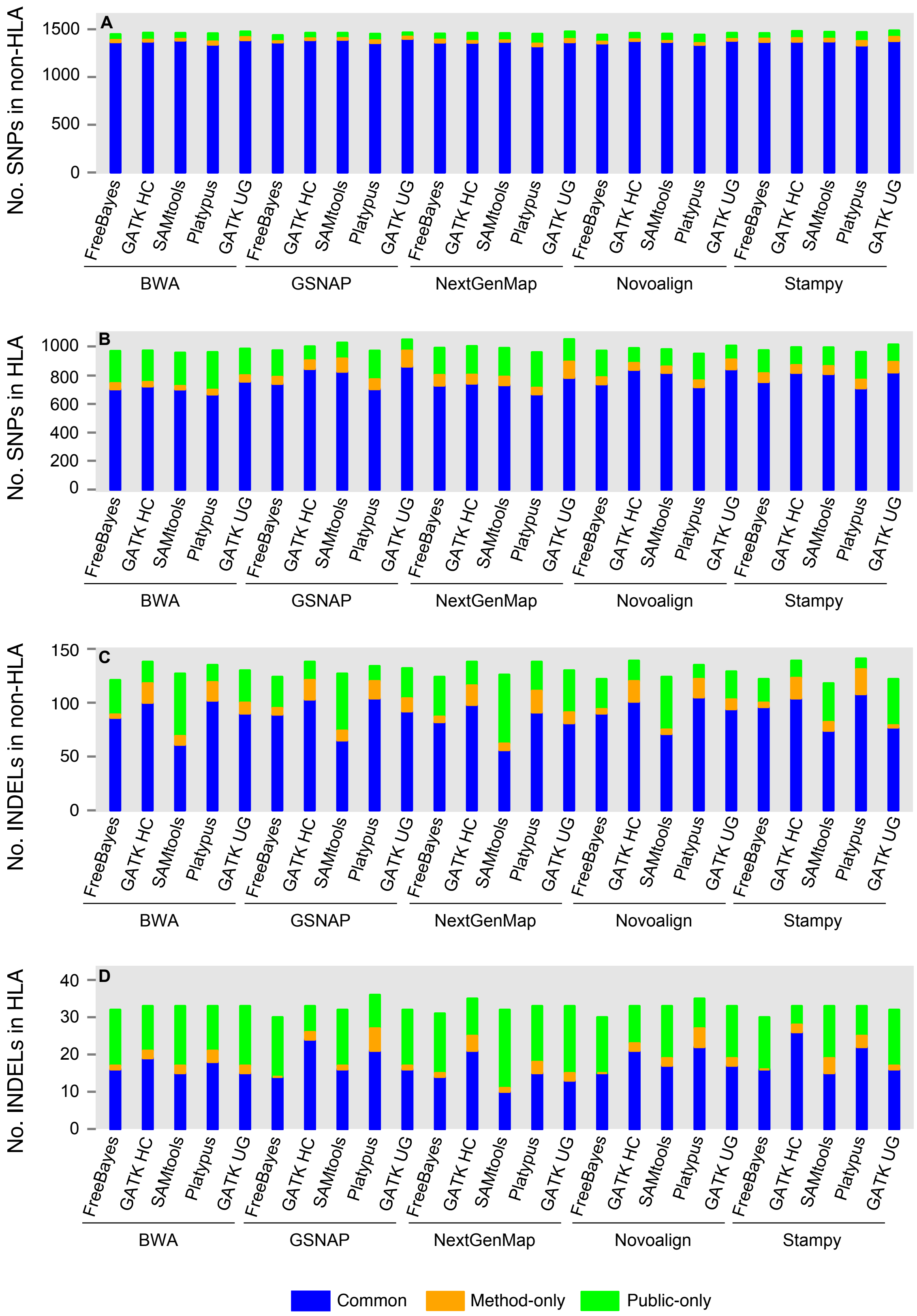
A
B
C
D
No. SNPs in non-HLA
No. SNPs in HLA
No. INDELs in non-HLA
No. INDELs in HLA
FreeBayes
GATK HC
SAMtools
Platypus
GATK UG
BWA
GSNAP
NextGenMap
Novoalign
Stampy
Common
Method-only
Public-only

Figure S4. Number of known variants in the HLA and non-HLA regions of Chr6 in NA12878. The first 100 bases from the 150-base exome-seq data in NA12878 (FC1_NA12878_01) were mapped by five aligners and variants were identified using five callers (*x*-axis). *Y*-axis shows the number of known SNPs (**A-B**) and INDELs (**C-D**) that match dbSNP v138. For both HLA (**B** and **D**) and non-HLA regions (**A** and **C**), known INDELs and SNPs are broken into: a common portion shared with the 'public call set' ('common', see below), and two portions unique to a given mapper-caller combination ('Method-only') and to the public call set ('Public-only'), respectively. Exome data FC1_NA12878_01 is one of the twelve replicates that are available at https://basespace.illumina.com/analyses/6847907/inputs. The public call set in NA12878 is the union of four call sets below. The high confident call set was generated from 11 whole genome and three exome sequencing datasets and is available at ftp://ftp-trace.ncbi.nih.gov/giab/ftp/data/NA12878/variant_calls/GIAB_integration/NIST_RTG_PlatGen_merged_highconfidence_v0.2.primitives.vcf.gz [9]. The other three lists of variants were called by three packages, Cortex, GATK HaplotypeCaller and DISCOVAR, from 250-bp paired-end reads generated from a PCR-free genome sequencing library (http://genepattern.broadinstitute.org/ftp/distribution/crd/DiscovarManuscript/vcf/) [10]. NA12878 exome-seq data refers to FC1_NA12878_01 (replicate 1) in all figures, unless stated otherwise. HLA region, 29,500,000-33,500,000 bp on Chr6; GATK HC, GATK HaplotypeCaller; GATK UG, GATK UnifiedGenotyper.

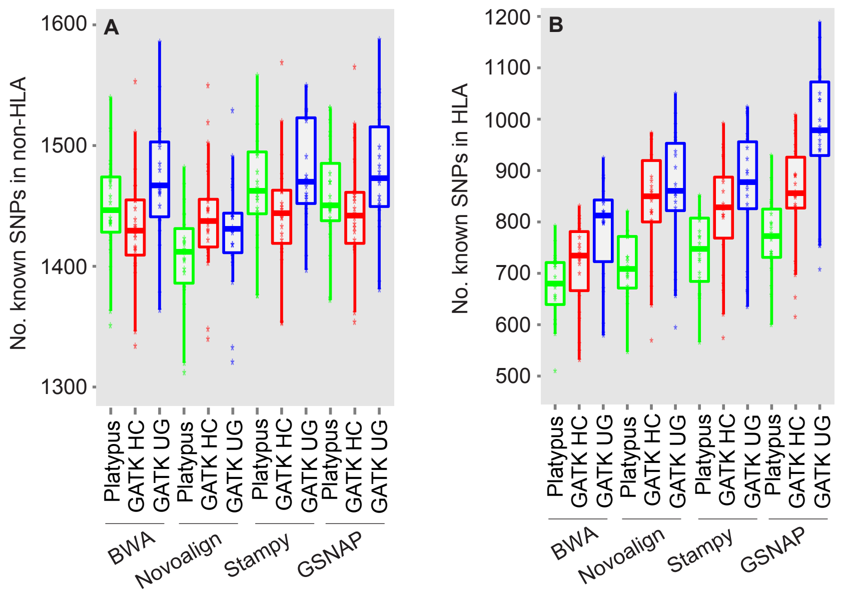

Figure S5. Number of known SNPs in the HLA and non-HLA regions of Chr6 in CLL. SNPs were called from 22 CLL samples using four mappers together with three callers. Known SNPs were identified by intersecting with dbSNP v138. (**A**) Non-HLA regions from Chr6. (**B**) HLA.

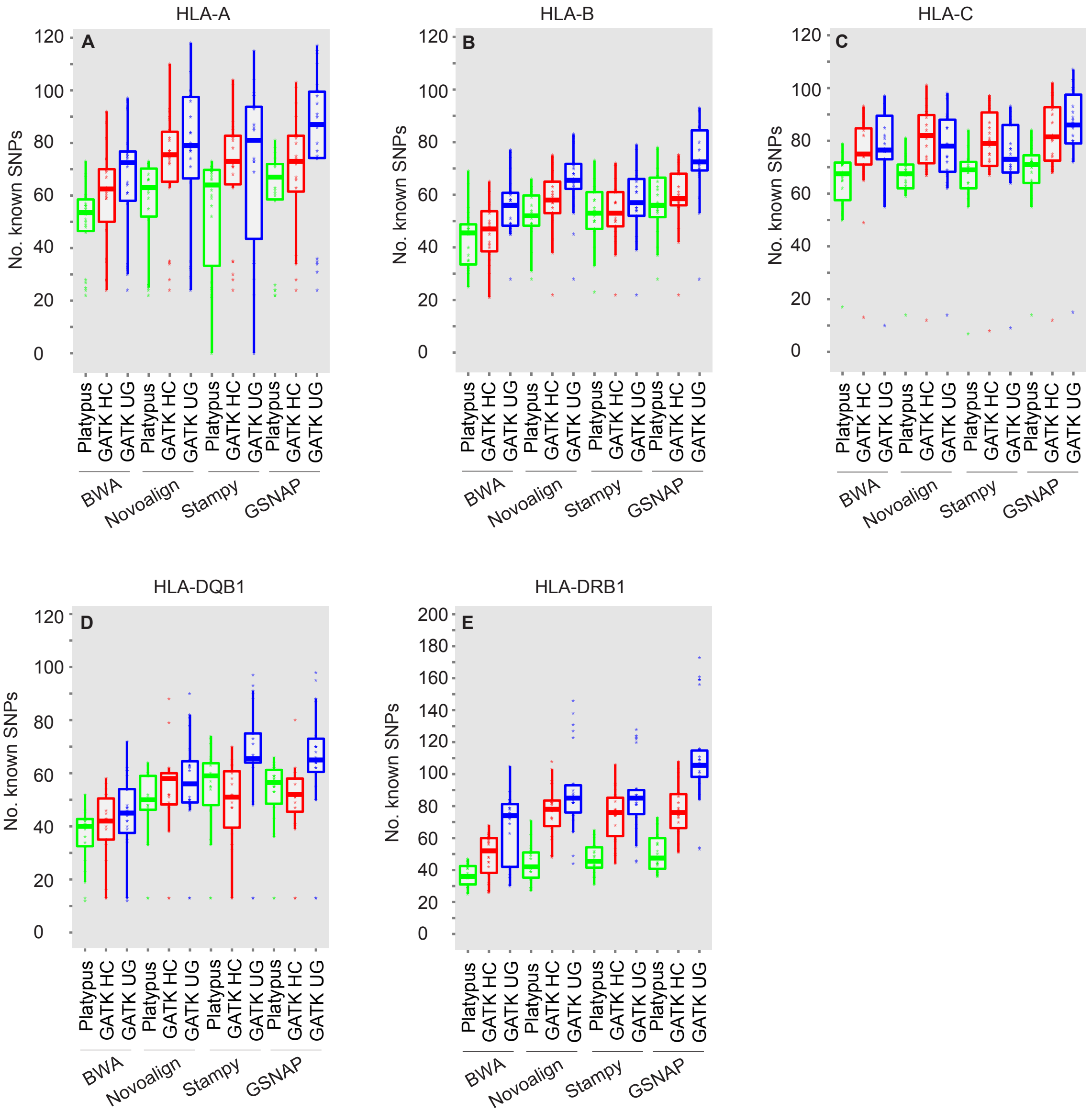

Figure S6. Number of known SNPs in five HLA genes. (**A-E**) Known SNPs identified in HLA-A (**A**), HLA-B (**B**), HLA-C (**C**), HLA-DQB1 (**D**) and HLA-DRB1 (**E**). See Figure S5 legend for details.

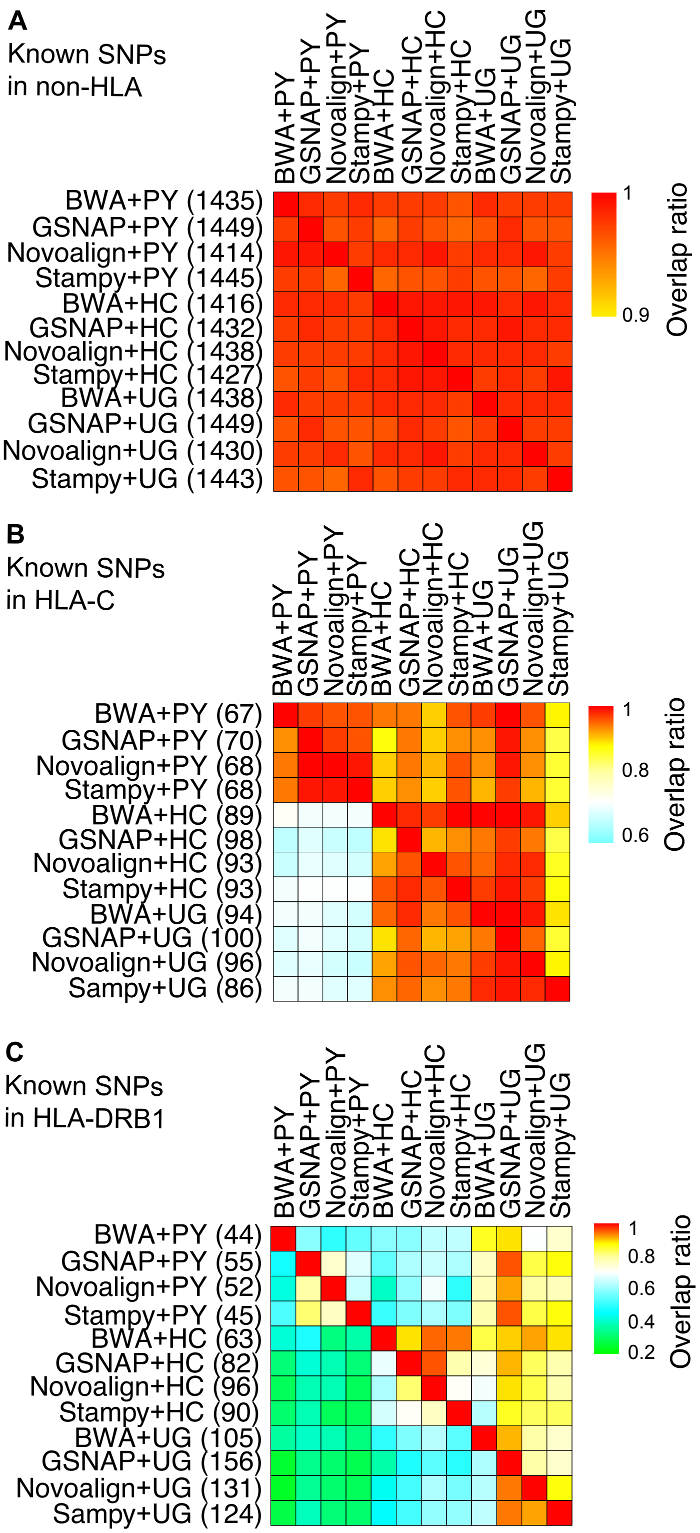

Figure S7. Heat maps illustrating the overlap of known SNPs. Variants were identified from the CLL sample 612703. (**A**) Known SNPs in the non-HLA regions of Chr6. (**B**) Known SNPs in HLA-C. (**C**) Known SNPs in HLA-DRB1. See Figure 4 legend for more information.

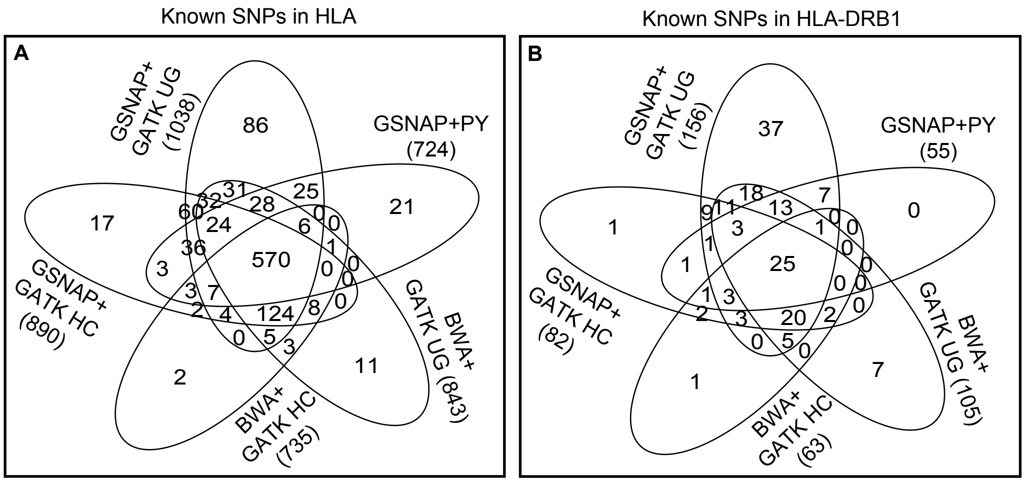

Figure S8. Venn diagrams depicting the overlap of known SNPs. SNPs were identified by GATK HaplotypeCaller (GATK HC), GATK UnifiedGenotyper (GATK UG) and Platypus from BWA and GSNAP mapped reads in the CLL sample 612703. Number of known SNPs matching dbSNP v138 are shown in parentheses. (**A**) HLA region. (**B**) HLA-DRB1.

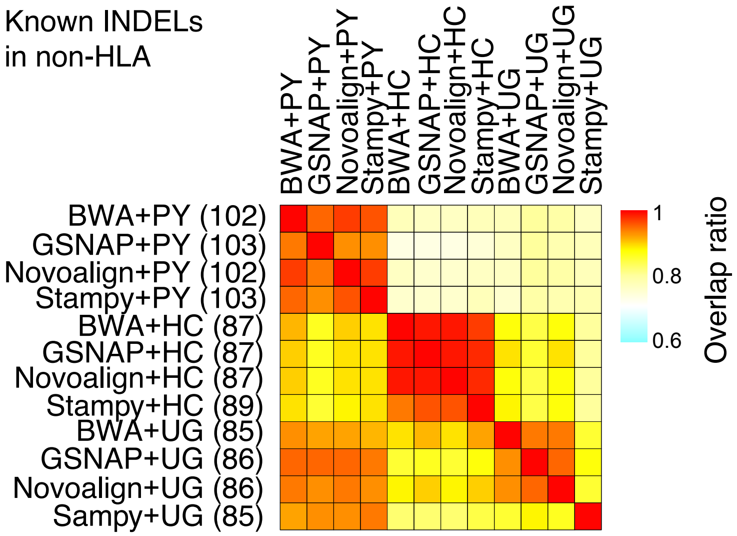

Figure S9. Overlap of known INDELs in the non-HLA regions of Chr6. INDELs were identified from the CLL sample 612703. Known INDELs are those that match dbSNP v138. See Figure 4 legend for more information.

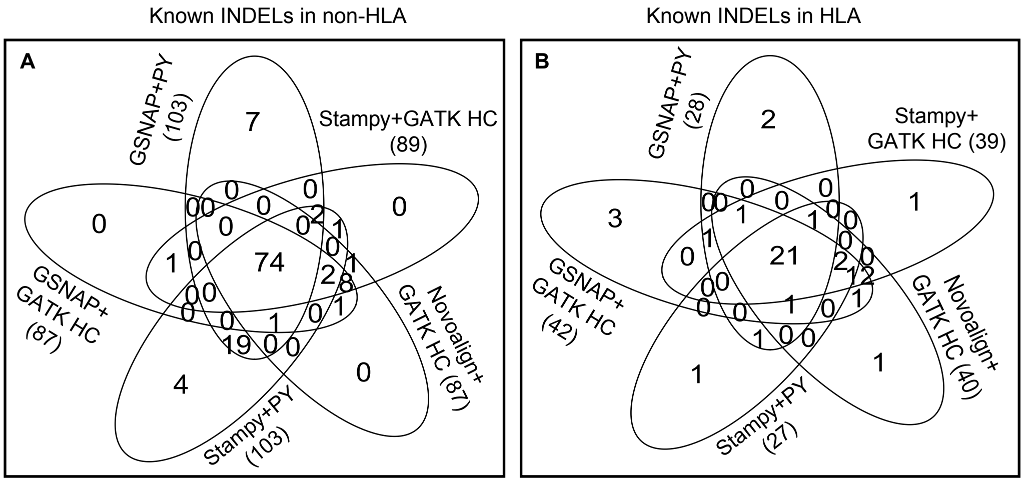

Figure S10. Venn diagrams depicting the overlap of known INDELs. INDELs were identified from the CLL sample 612703, using GATK HaplotypeCaller (GATK HC) and Platypus (PY) together with GSNAP, Novoalign and Stampy mapping. Known INDELs are those that match dbSNP v138 and are shown in parentheses. (**A**) Non-HLA regions of Chr6. (**B**) HLA region.

References

1. Li H, Durbin R. Fast and accurate short read alignment with Burrows-Wheeler transform. Bioinformatics*.* 2009;25(14):1754-60.
2. Wu TD, Nacu S. Fast and SNP-tolerant detection of complex variants and splicing in short reads. Bioinformatics*.* 2010;26(7):873-81.
3. Sedlazeck FJ, Rescheneder P, von Haeseler A. NextGenMap: fast and accurate read mapping in highly polymorphic genomes. Bioinformatics*.* 2013;29(21):2790-1.
4. Lunter G, Goodson M. Stampy: a statistical algorithm for sensitive and fast mapping of Illumina sequence reads. Genome Res*.* 2011;21(6):936-9.
5. Li H, Handsaker B, Wysoker A, Fennell T, Ruan J, Homer N, Marth G, Abecasis G, Durbin R. The Sequence Alignment/Map format and SAMtools. Bioinformatics*.* 2009;25(16):2078-9.

6. DePristo MA, Banks E, Poplin R, Garimella KV, Maguire JR, Hartl C, Philippakis AA, del Angel G, Rivas MA, Hanna M *et al*. A framework for variation discovery and genotyping using next-generation DNA sequencing data. Nat Genet. 2011;43(5):491-8.
7. Rimmer A, Phan H, Mathieson I, Iqbal Z, Twigg SR, Wilkie AO, McVean G, Lunter G. Integrating mapping-, assembly- and haplotype-based approaches for calling variants in clinical sequencing applications. Nat Genet. 2014;46(8):912-8.
8. Quinlan AR, Hall IM. BEDTools: a flexible suite of utilities for comparing genomic features. Bioinformatics. 2010;26(6):841-2.
9. Zook JM, Chapman B, Wang J, Mittelman D, Hofmann O, Hide W, Salit M. Integrating human sequence data sets provides a resource of benchmark SNP and indel genotype calls. Nat Biotechnol. 2014;32(3):246-51.
10. Weisenfeld NI, Yin S, Sharpe T, Lau B, Hegarty R, Holmes L, Sogoloff B, Tabbaa D, Williams L, Russ C *et al*. Comprehensive variation discovery in single human genomes. Nat Genet. 2014;46(12):1350-5.
