# Supplementary material for: An analytical workflow for accurate variant discovery in highly divergent regions
Source: BMC Genomics. 2016 Sep 2;17(1):703. doi: 10.1186/s12864-016-3045-z (PMC5010666; doi:10.1186/s12864-016-3045-z)
Supplement: Additional file 2: Table S1. — Five mappers and five variant callers used in the study. Table S2. SNP and INDEL calling precision rate in simulated data. Table S3. Percent of SNP calling sensitivity for three callers in simulated data. Table S4. INDEL calling sensitivity for two callers in simulated data. Table S5. Known INDELs in the HLA region of NA12878. Table S6. Novel INDELs in the HLA region of NA12878. Table S7. Number of novel SNPs in 22 CLL samples. Table S8. Number of INDELs in 22 CLL samples. (PDF 170 kb) [file 12864_2016_3045_MOESM2_ESM.pdf]

Additional file 2

Table S1. Five mappers and five variant callers used in the study

Tool	Version	Command line	Reference
BWA	v0.5.9	1) bwa index ref.fa; 2) bwa aln ref.fa end1.fastq > end1.sai; 3) bwa aln ref.fa end2.fastq > end2.sai; 4) bwa sampe -0 1000 -f out.sam ref.fa end1.sai end2.sai end1.fastq end2.fastq	[1]
Novoalign	v3.01.01	1) novoindex ref.nix ref.fa; 2) novoalign --hdrhd off -i PE 425,80 -r Random -F STDFQ -v 90 -x 5 -o SAM -d ref.nix -f end1.fastq end2.fastq > out.sam	www.novocraft.com
Stampy	v1.0.21	1) stampy.py --species=human --assembly=hg19_ncbi37 -G ref.fa; 2) stampy.py -g ref -H ref; 3a) stampy.py -g ref -h ref --substitutionrate=0.1 -f sam --inputformat=fastq -o out.sam -M end1.fastq end2.fastq (for simulated data); 3b) stampy.py -g ref -h ref --substitutionrate=0.1 -f sam --inputformat=fastq --solexa --xa-max=3 --xa-max-discordant=10 -o out.sam -M end1.fastq end2.fastq (for real data)	[2]
GSNAP	v2013-10-25	1) gmap_build -d ref.gmapdb -k 13 ref.fa; 2) gsnap -d ref.gmapdb -D ref.gmapdb -k 13 --orientation FR --max-mismatches 0.1 --maxsearch 1 --npaths 1 --ordered --show-refdiff -A sam end1.fastq end2.fastq > out.sam	[3]
NextGenMap	v0.4.9	1) ngm -r ref.fa -o ref.ngm; 2) ngm -r ref.fa -1 end1.fastq -2 end2.fastq -k 15 -I 0 -X 800 -i 0.8 -n 1 -p -o out.sam	[4]
GATK UnifiedGenotyper	v2.7-2	GenomeAnalysisTK.jar -T UnifiedGenotyper -L chr6 -isr intersection -glm BOTH -mbq 17 --dbsnp dbsnp_135.hg19.vcf.gz -stand_call_conf 20 -stand_emit_conf 10	[5, 6]
GATK HaplotypeCaller	v2.7-2	GenomeAnalysisTK.jar -T HaplotypeCaller -L chr6 -isr intersection --genotyping_mode DISCOVERY --dbsnp dbsnp_135.hg19.vcf.gz -stand_call_conf 20 -stand_emit_conf 10	[5, 6]

FreeBayes	v9.9.2-27	freebayes -b in.bam -f ref.fa -t target.bed -C 2 -3 40 -P 0.0001 -m 0 -q 17 -W 1,3 -S 4 -M 3 -B 25 -E 3 -v out.vcf	[7]
SAMtools mpileup	v0.1.19	samtools mpileup -q 0 -Q 17 -B -ugf ref.fa -l target.bed -F 0.002 in.bam | bcftools view -bvcg - > out.bcf; 2) bcftools view out.bcf >out.vcf	[8]
Platypus	v0.5.2	Platypus.py --bamFiles=in.bam --refFile=ref.fa --maxVariants=50 minMapQual=0 --rmsmqThreshold=0 --hapScoreThreshold=0 --minBaseQual=17 --regions=target.bed --output=out.vcf	[9]

ref.fa, hg19 reference sequence in fasta format.
target.bed, a list of non-overlapping Chr6 regions used in simulation or a list of 'on-target' regions for real exome-seq data, see Methods section for details.
in.bam, mapping output BAM file which has gone through coordinate-based sorting, duplicate marking and local realignment.

Table S2. SNP and INDEL calling precision rate in simulated data

Type	Div (%)	Cov	Caller				
			GATK UG	Platypus	SAMtools	GATK HC	FreeBayes
SNP	<=1	10	99.0-100	98.2-100	99.3-100	99.1-100	98.3-99.6
SNP	<=1	40-100	98.0-100	96.6-100	98.9-100	97.8-100	98.1-99.6
SNP	5-10	10	98.9-99.7	97.5-99.3	98.8-99.8	99.3-99.6	99.0-99.3
SNP	5-10	40-100	98.5-99.7	92.3-98.9	98.7-99.8	99.2-99.6	98.9-99.3
INDEL	<=1	10	99.3-100	98.5-99.9	99.5-100	99.3-99.9	98.0-99.8
INDEL	<=1	40-100	99.0-100	98.3-99.9	99.0-100	98.7-99.9	98.7-99.9
INDEL	5-10	10	93.5-99.7	89.1-99.1	95.2-99.7	92.6-97.8	94.1-99.1
INDEL	5-10	40-100	87.9-99.5	89.7-99.0	78.4-99.5	92.5-97.8	93.8-99.0

The values are the range of the percent of precision rate for each caller, calculated from the associated mappers and divergence levels. GATK UG, GATK UnifiedGenotyper; GATK HC, GATK HaplotypeCaller; Div, divergence; Cov, coverage.

Table S3. Percent of SNP calling sensitivity for three callers in simulated data

Caller	Div	Cov	Mapper				
	(%)		BWA	GSNAP	NGM	Novoalign	Stampy
SAMtools	<=1	10	87.5-88.4	86.4-87.4	86.0-87.0	85.9-86.8	87.2-87.9
SAMtools	<=1	40	96.3-96.8	95.8-96.5	95.3-96.1	95.5-96.1	96.1-96.6
SAMtools	<=1	100	97.7-98.2	97.2-97.9	96.7-97.5	96.9-97.7	97.4-98.1
SAMtools	5-10	10	-	84.0-85.2	80.7-83.4	81.9-84.3	83.9-85.5
SAMtools	5-10	40	-	93.4-93.9	90.8-92.5	91.7-93.3	91.6-93.5
SAMtools	5-10	100	-	94.5-95.1	91.9-93.8	92.9-94.5	90.9-94.1
SAMtools	<=1	10	A	C	C	C	B
SAMtools	<=1	40,100	A	B	B	B	A
SAMtools	5-10	10	-	A	C	B	A
SAMtools	5-10	40,100	-	A	C	B	B
GATK HC	<=1	10	86.5-87.3	86.0-87.0	85.4-86.6	85.8-86.8	86.1-86.6
GATK HC	<=1	40	95.9-96.3	96.0-96.4	95.3-95.8	95.9-96.3	95.7-96.0
GATK HC	<=1	100	96.8-97.2	96.9-97.4	96.4-96.7	96.9-97.4	96.8-97.0
GATK HC	5-10	10	-	78.1-84.0	73.6-81.7	78.7-84.0	78.2-83.6
GATK HC	5-10	40	-	89.5-94.0	85.7-92.0	89.6-94.0	89.5-93.8
GATK HC	5-10	100	-	90.7-95.3	87.3-93.5	90.9-95.2	90.9-95.1
GATK HC	<=1	10	A	B	B	B	B
GATK HC	<=1	40,100	A	A	C	A	B
GATK HC	5-10	10	-	A	B	A	A
GATK HC	5-10	40,100	-	A	B	A	A
GATK UG	<=1	10	89.2-89.8	87.8-88.8	87.3-88.5	87.4-88.3	88.8-89.1
GATK UG	<=1	40	97.1-97.3	96.4-96.9	96.0-96.3	96.2-96.6	96.9-97.2
GATK UG	<=1	100	98.2-98.5	97.5-98.2	97.3-97.6	97.3-98.0	98.1-98.4
GATK UG	5-10	10	-	86.2-88.0	82.2-85.8	84.0-87.0	83.2-87.5
GATK UG	5-10	40	-	95.1-96.2	91.9-94.4	93.4-95.6	91.3-95.5
GATK UG	5-10	100	-	96.4-97.4	93.5-95.8	94.7-96.8	92.7-96.8
GATK UG	<=1	10	A	C	C	C	B
GATK UG	<=1	40,100	A	B	B	B	A
GATK UG	5-10	10	-	A	C	B	B
GATK UG	5-10	40,100	-	A	C	B	C

Shown are the range and rank (A to C) of the percent of SNP calling sensitivity for each method. The datasets are split into four groups based on coverage and divergence, as in **Table 1**. For each caller within each group, the associated mappers are ranked based on the sensitivity, with "A" indicating the mapper with the highest overall sensitivity together with a given caller. BWA mapping results at 5-10% divergence were not shown. GATK UG, GATK UnifiedGenotyper; GATK HC, GATK HaplotypeCaller; Div, divergence; Cov, coverage; NGM, NextGenMap.

Table S4. INDEL calling sensitivity for two callers in simulated data

Caller	Div (%)	Cov	Mapper				
			BWA	GSNAP	NGM	Novoalign	Stampy
Platypus	<=1	10	70.5-74.5	69.1-73.1	70.7-74.2	69.7-72.7	70.8-74.9
Platypus	<=1	40	78.3-81.8	77.8-82.2	79.5-83.3	78.1-82.2	78.6-82.5
Platypus	<=1	100	79.5-83.3	78.8-83.3	81.0-85.5	79.2-83.6	79.7-84.0
Platypus	5-10	10	-	64.1-68.4	68.3-70.9	64.5-68.9	67.4-69.8
Platypus	5-10	40	-	72.8-76.3	77.7-80.1	73.9-77.2	75.2-77.5
Platypus	5-10	100	-	74.2-77.5	79.0-81.7	75.1-78.3	76.2-78.6
Platypus	<=1	10	B	C	B	C	A
Platypus	<=1	40,100	B	B	A	B	B
Platypus	5-10	10	-	C	A	C	B
Platypus	5-10	40,100	-	D	A	C	B
GATK HC	<=1	10	70.1-74.5	70.2-74.2	69.7-74.2	70.2-74.2	69.8-74.5
GATK HC	<=1	40	77.9-81.1	78.0-81.1	77.9-81.1	78.0-81.1	77.9-81.1
GATK HC	<=1	100	78.7-82.9	78.7-82.9	79.0-83.3	78.7-82.9	78.7-82.9
GATK HC	5-10	10	-	63.9-69.0	58.4-66.0	64.6-68.9	64.3-68.6
GATK HC	5-10	40	-	74.4-77.4	70.8-75.8	74.4-77.4	74.5-77.2
GATK HC	5-10	100	-	75.5-78.6	72.4-77.5	75.6-78.5	75.8-78.5
GATK HC	<=1	10	A	A	B	A	A
GATK HC	<=1	40,100	A	A	A	A	A
GATK HC	5-10	10	-	A	B	A	A
GATK HC	5-10	40,100	-	A	B	A	A

See **Table S3** for details. GATK HC, GATK HaplotypeCaller; Div, divergence; Cov, coverage; NGM, NextGenMap.

Table S5. Known INDELs in the HLA region of NA12878

Rep	Caller	No. INDELs from four mappers						Public call set		
		BWA	GSNAP	Novoalign	Stampy	Total		250-bp	Conf	Total
1	GATK HC	0	0	0	0	5		5	0	5
1	GATK HC	0	0	0	3	3		2	1	2
1	GATK HC	0	3	0	3	3		3	0	3
1	GATK HC	0	2	2	2	2		2	0	2
1	GATK HC	1	1	1	0	1		0	0	0
1	GATK HC	20	20	20	20	20		19	5	19
1	Sub total	21	26	23	28	34		31	6	31
2	GATK HC	0	0	0	0	4		4	0	4
2	GATK HC	0	0	0	3	3		3	0	3
2	GATK HC	0	2	0	0	2		0	0	0
2	GATK HC	0	2	2	2	2		2	0	2
2	GATK HC	26	26	26	26	26		22	6	22
2	Sub total	26	30	28	31	37		31	6	31
1	Platypus	0	0	0	0	7		7	0	7
1	Platypus	0	0	0	2	2		1	0	1
1	Platypus	0	1	0	0	1		0	0	0
1	Platypus	0	3	3	0	3		0	0	0
1	Platypus	0	4	4	4	4		4	0	4
1	Platypus	1	0	0	0	1		0	0	0
1	Platypus	1	0	1	0	1		1	0	1
1	Platypus	19	19	19	19	19		17	6	17
1	Sub total	21	27	27	25	38		30	6	30
2	Platypus	0	0	0	0	3		3	0	3
2	Platypus	0	0	0	3	3		2	0	2
2	Platypus	0	2	0	0	2		0	0	0
2	Platypus	0	1	1	0	1		0	0	0
2	Platypus	0	4	4	4	4		4	0	4
2	Platypus	3	0	0	0	3		1	0	1
2	Platypus	1	0	1	1	1		1	0	1
2	Platypus	1	1	0	1	1		0	0	0
2	Platypus	21	21	21	21	21		19	6	19
2	Sub total	26	29	27	30	39		30	6	30

Shown is the number of known INDELs in the HLA region that are identified by this study or present in the public call set. Public call set is combined from Cortex, DISCOVAR and GATK HaplotypeCaller calls from 250-bp paired sequencing of a PCR-free genomic library (‘250-bp’) [10] and a high-confident call set (‘Conf’) in NA12878 [11]. GATK HC, GATK HaplotypeCaller; HLA, Chr6:29,500,000-33,500,000 bp.

Table S6. Novel INDELs in the HLA region of NA12878

Rep	Caller	No. INDELs from four mappers						Public call set		
		BWA	GSNAP	Novoalign	Stampy	Total		250-bp	Conf	Total
1	GATK HC	0	0	0	0	19		18	1	19
1	GATK HC	0	0	0	3	3		2	0	2
1	GATK HC	0	2	0	0	2		0	0	0
1	GATK HC	0	1	0	1	1		0	0	0
1	GATK HC	0	3	3	0	3		3	0	3
1	GATK HC	0	8	8	8	8		8	0	8
1	GATK HC	2	0	0	0	2		1	0	1
1	GATK HC	2	2	2	0	2		1	0	1
1	GATK HC	24	24	24	24	24		19	5	19
1	Sub total	28	40	37	36	64		52	6	53
2	GATK HC	0	0	0	0	13		13	1	13
2	GATK HC	0	0	0	7	7		6	0	6
2	GATK HC	0	0	2	0	2		0	0	0
2	GATK HC	0	1	0	1	1		1	0	1
2	GATK HC	0	2	2	0	2		2	0	2
2	GATK HC	0	14	14	14	14		9	0	9
2	GATK HC	1	0	0	1	1		0	0	0
2	GATK HC	1	0	1	0	1		1	0	1
2	GATK HC	1	1	1	0	1		0	0	0
2	GATK HC	22	22	22	22	22		20	5	20
2	Sub total	25	40	42	45	64		52	6	52
1	Platypus	0	0	0	0	36		36	1	36
1	Platypus	0	0	0	14	14		5	0	5
1	Platypus	0	0	4	0	4		0	0	0
1	Platypus	0	0	1	1	1		0	0	0
1	Platypus	0	2	0	0	2		0	0	0
1	Platypus	0	2	2	2	2		1	0	1
1	Platypus	2	0	0	0	2		1	0	1
1	Platypus	1	0	1	0	1		0	0	0
1	Platypus	1	1	0	1	1		0	0	0
1	Platypus	11	11	11	11	11		10	5	10
1	Sub total	15	16	19	29	74		53	6	53
2	Platypus	0	0	0	0	25		25	1	25
2	Platypus	0	0	0	21	21		12	0	12
2	Platypus	0	0	5	5	5		1	0	1
2	Platypus	0	3	0	0	3		0	0	0
2	Platypus	0	1	0	1	1		1	0	1
2	Platypus	0	1	1	1	1		1	0	1
2	Platypus	2	0	0	0	2		1	0	1
2	Platypus	1	0	0	1	1		1	0	1
2	Platypus	1	0	1	1	1		0	0	0
2	Platypus	4	4	0	0	4		1	0	1
2	Platypus	12	12	12	12	12		10	5	10
2	Sub total	20	21	19	42	76		53	6	53

Shown is the number of novel INDELs (not in dbSNP v138) in the HLA region. See **Table S5** for details.

Table S7. Number of novel SNPs in 22 CLL samples

Mapper	Caller	Target	
		HLA	Non-HLA
BWA	GATK HC	33 (17-48)	31 (3-67)
GSNAP	GATK HC	64 (21-99)	46 (22-91)
Novoalign	GATK HC	42 (19-82)	34 (6-80)
Stampy	GATK HC	55 (22-102)	74 (43-108)
BWA	GATK UG	32 (20-59)	36 (6-157)
GSNAP	GATK UG	78 (30-107)	46 (24-153)
Novoalign	GATK UG	32 (19-42)	11 (5-43)
Stampy	GATK UG	62 (25-102)	92 (45-239)
BWA	Platypus	32 (23-76)	86 (23-195)
GSNAP	Platypus	54 (33-86)	76 (34-211)
Novoalign	Platypus	31 (22-49)	26 (9-69)
Stampy	Platypus	50 (30-93)	156 (65-351)

Shown are the median and range (in parentheses) of novel SNPs not matching dbSNP v138. HC, GATK HaplotypeCaller; UG, GATK UnifiedGenotyper. HLA, Chr6:29,500,000-33,500,000 bp; Non-HLA, other capture regions from Chr6.

Table S8. Number of INDELs in 22 CLL samples

Mapper	Caller	Target			
		HLA (known)	HLA (novel)	Non-HLA (known)	Non-HLA (novel)
BWA	GATK HC	28 (19-35)	26 (16-38)	98 (81-114)	26 (19-35)
GSNAP	GATK HC	36 (28-49)	42 (26-58)	99 (82-110)	27 (18-38)
Novoalign	GATK HC	36 (25-45)	37 (19-52)	98 (83-112)	27 (21-36)
Stampy	GATK HC	34 (25-47)	36 (23-53)	98 (80-110)	24 (18-34)
BWA	GATK UG	20 (12-27)	10 (5-14)	98 (83-110)	10 (3-18)
GSNAP	GATK UG	24 (16-33)	11 (5-14)	98 (84-113)	8 (2-13)
Novoalign	GATK UG	24 (18-30)	13 (7-19)	98 (85-111)	10 (5-16)
Stampy	GATK UG	19 (10-28)	32 (14-43)	86 (73-104)	21 (14-33)
BWA	Platypus	26 (21-37)	14 (10-19)	110 (95-129)	15 (7-35)
GSNAP	Platypus	28 (22-37)	16 (10-21)	111 (97-126)	11 (7-32)
Novoalign	Platypus	30 (21-38)	22 (11-31)	110 (97-126)	9 (4-14)
Stampy	Platypus	27 (18-36)	40 (25-53)	111 (98-128)	22 (11-236)

Shown are the median and range (in parentheses) of INDELs called from 22 CLL samples. Known, INDEL matching dbSNP v138; novel, INDEL not matching dbSNP v138. GATK HC, GATK HaplotypeCaller; GATK UG, GATK UnifiedGenotyper. HLA, Chr6:29,500,000-33,500,000 bp; Non-HLA, other capture regions from Chr6.

References

1. Li H, Durbin R. Fast and accurate short read alignment with Burrows-Wheeler transform. Bioinformatics. 2009;25(14):1754-60.
2. Lunter G, Goodson M. Stampy: a statistical algorithm for sensitive and fast mapping of Illumina sequence reads. Genome Res. 2011;21(6):936-9.
3. Wu TD, Nacu S. Fast and SNP-tolerant detection of complex variants and splicing in short reads. Bioinformatics. 2010;26(7):873-81.

4. Sedlazeck FJ, Rescheneder P, von Haeseler A. NextGenMap: fast and accurate read mapping in highly polymorphic genomes. Bioinformatics. 2013;29(21):2790-1.
5. McKenna A, Hanna M, Banks E, Sivachenko A, Cibulskis K, Kernytsky A, Garimella K, Altshuler D, Gabriel S, Daly M *et al*. The Genome Analysis Toolkit: a MapReduce framework for analyzing next-generation DNA sequencing data. Genome Res. 2010;20(9):1297-303.
6. DePristo MA, Banks E, Poplin R, Garimella KV, Maguire JR, Hartl C, Philippakis AA, del Angel G, Rivas MA, Hanna M *et al*. A framework for variation discovery and genotyping using next-generation DNA sequencing data. Nat Genet. 2011;43(5):491-8.
7. Garrison E, Marth G. Haplotype-based variant detection from short-read sequencing. http://arxiv.org/abs/1207.3907v2. 2012.
8. Li H, Handsaker B, Wysoker A, Fennell T, Ruan J, Homer N, Marth G, Abecasis G, Durbin R. The Sequence Alignment/Map format and SAMtools. Bioinformatics. 2009;25(16):2078-9.
9. Rimmer A, Phan H, Mathieson I, Iqbal Z, Twigg SR, Wilkie AO, McVean G, Lunter G. Integrating mapping-, assembly- and haplotype-based approaches for calling variants in clinical sequencing applications. Nat Genet. 2014;46(8):912-8.
10. Weisenfeld NI, Yin S, Sharpe T, Lau B, Hegarty R, Holmes L, Sogoloff B, Tabbaa D, Williams L, Russ C *et al*. Comprehensive variation discovery in single human genomes. Nat Genet. 2014;46(12):1350-5.
11. Zook JM, Chapman B, Wang J, Mittelman D, Hofmann O, Hide W, Salit M. Integrating human sequence data sets provides a resource of benchmark SNP and indel genotype calls. Nat Biotechnol. 2014;32(3):246-51.
